# Decreased bone formation and increased osteoclastogenesis cause bone loss in mucolipidosis II

**DOI:** 10.1002/emmm.201302979

**Published:** 2013-10-15

**Authors:** Katrin Kollmann, Jan Malte Pestka, Sonja Christin Kühn, Elisabeth Schöne, Michaela Schweizer, Kathrin Karkmann, Takanobu Otomo, Philip Catala-Lehnen, Antonio Virgilio Failla, Robert Percy Marshall, Matthias Krause, Rene Santer, Michael Amling, Thomas Braulke, Thorsten Schinke

**Affiliations:** 1 Department of Biochemistry, Children's Hospital, University Medical Center Hamburg-EppendorfHamburg, Germany; 2 Department of Osteology and Biomechanics, Center of Experimental Medicine, University Medical Center Hamburg-EppendorfHamburg, Germany; 3 Department of Electron Microscopy, Center for Molecular Neurobiology, University Medical Center Hamburg-EppendorfHamburg, Germany; 4 UKE Microscopy Imaging Facility, University Medical Center Hamburg-EppendorfHamburg, Germany

**Keywords:** alendronate, interleukin-6, mannose 6-phosphate, mucolipidosis II, osteoclastogenesis

## Abstract

Mucolipidosis type II (MLII) is a severe multi-systemic genetic disorder caused by missorting of lysosomal proteins and the subsequent lysosomal storage of undegraded macromolecules. Although affected children develop disabling skeletal abnormalities, their pathogenesis is not understood. Here we report that MLII knock-in mice, recapitulating the human storage disease, are runted with accompanying growth plate widening, low trabecular bone mass and cortical porosity. Intralysosomal deficiency of numerous acid hydrolases results in accumulation of storage material in chondrocytes and osteoblasts, and impaired bone formation. In osteoclasts, no morphological or functional abnormalities are detected whereas osteoclastogenesis is dramatically increased in MLII mice. The high number of osteoclasts in MLII is associated with enhanced osteoblastic expression of the pro-osteoclastogenic cytokine interleukin-6, and pharmacological inhibition of bone resorption prevented the osteoporotic phenotype of MLII mice. Our findings show that progressive bone loss in MLII is due to the presence of dysfunctional osteoblasts combined with excessive osteoclastogenesis. They further underscore the importance of a deep skeletal phenotyping approach for other lysosomal diseases in which bone loss is a prominent feature.

## INTRODUCTION

Targeting of soluble lysosomal hydrolases requires mannose 6-phosphate (M6P) residues formed by the sequential action of the Golgi-resident *N*-acetylglucosamine (GlcNAc)-1-phosphotransferase complex consisting of three subunits (α_2_β_2_γ_2_), and the GlcNAc-1-phosphodiester α-*N*-acetylglucosaminidase (‘uncovering enzyme’) (Bao et al, 1996; Braulke et al, 2013; Kollmann et al, 2010). The α and β subunits encoded by *GNPTAB* (Kudo et al, 2005; Tiede et al, 2005) are synthesized as a type III membrane precursor protein that is activated proteolytically by site-1 protease (Marschner et al, 2011). The function of the soluble γ subunit, encoded by the *GNPTG* gene (Raas-Rothschild et al, 2000), is unknown. Mutations in *GNPTAB* can result in mucolipidosis type II (MLII alpha/beta, I-cell disease) clinically characterized by impaired skeletal growth, progressive osteodystrophy, destructive bone lesions (‘dysostosis multiplex’), facial dysmorphism, psychomotor retardation, cardiorespiratory defects and early death between 5 and 8 years of age (Braulke et al, 2013; Cathey et al, 2010; Spranger & Wiedemann, 1970). The majority of these severely affected MLII patients have nonsense, frameshift or splice site alterations in *GNPTAB*. In addition, there are patients carrying missense mutations in *GNPTAB* (MLIII alpha/beta; pseudo-Hurler polydystrophy) resulting in residual GlcNAc-1-phosphotransferase activity and a milder course of disease with a later onset of clinical signs and symptoms, permitting survival into adulthood (Bargal et al, 2006; Braulke et al, 2013; Cathey et al, 2008; Cathey et al, 2010). The total loss of phosphotransferase activity causes missorting and hypersecretion of lysosomal hydrolases due to their inability to bind to M6P-specific receptors mediating the transport of hydrolases to lysosomes (Braulke & Bonifacino, 2009). The intracellular deficiency of multiple lysosomal hydrolases results in lysosomal dysfunction and accumulation of non-degraded material.

Bone development and remodelling requires a coordinated balance between bone-forming osteoblasts and bone-resorbing osteoclasts (Raggatt & Partridge, 2010). Osteoclasts are multinucleated cells formed by fusion of haematopoietic precursors of the monocyte/macrophage lineage undergoing a hormone and cytokine-dependent proliferation, differentiation and maturation process (Edwards & Mundy, 2011). The two most important physiological regulators of bone resorption are the pro-osteoclastogenic cytokine Rankl (receptor activator of nuclear factor κB ligand) and its antagonistically acting decoy receptor Opg (osteoprotegerin), which are predominantly expressed by osteoblasts. Parathyroid hormone (PTH) represents another osteoclastogenic regulator that affects bone resorption indirectly by increasing Rankl production in osteoblasts, and highlights the significance of a molecular communication between the two bone remodelling cell types. In the process of bone resorption a sealed and isolated resorptive microenvironment, the resorption lacuna, is formed between the osteoclasts and underlying bone matrix. Vacuolar-type proton pump, proton-chloride exchanger, and the lysosomal enzymes cathepsin K (CtsK) and tartrate-resistant acid phosphatase (TRAP, Acp5) comprise essential components of the resorptive machinery localized in secretory lysosomes (Coxon & Taylor, 2008) which fuse with the bone-apposing plasma membrane, the ruffled border, and release protons and lysosomal enzymes. Systematic analyses of cellular functions leading to skeletal defects in MLII are still missing because of the low incidence of the disease and early death of the patients. Since osteoclasts contain secretory lysosomes (Edwards & Mundy, 2011), it has been postulated that the hypersecretion of lysosomal hydrolases lacking M6P residues into the acidic resorption lacuna is responsible for increased bone resorption in MLII (van Meel et al, 2011). To analyse the impact of GlcNAc-1-phosphotransferase deficiency on skeletal alterations *in vivo*, we analysed phosphotransferase-defective mice generated by single base insertion into the *Gnptab* gene corresponding to a mutation detected in an MLII patient (Kollmann et al, 2012). Here we show that increased formation of intact osteoclasts, combined with decreased activity of bone-forming osteoblasts, is responsible for low bone mass in MLII mice, and that this phenotype can be corrected by anti-resorptive bisphosphonate treatment.

## RESULTS

### MLII mice are reduced in size and display osteopenia

**Figure 1:**
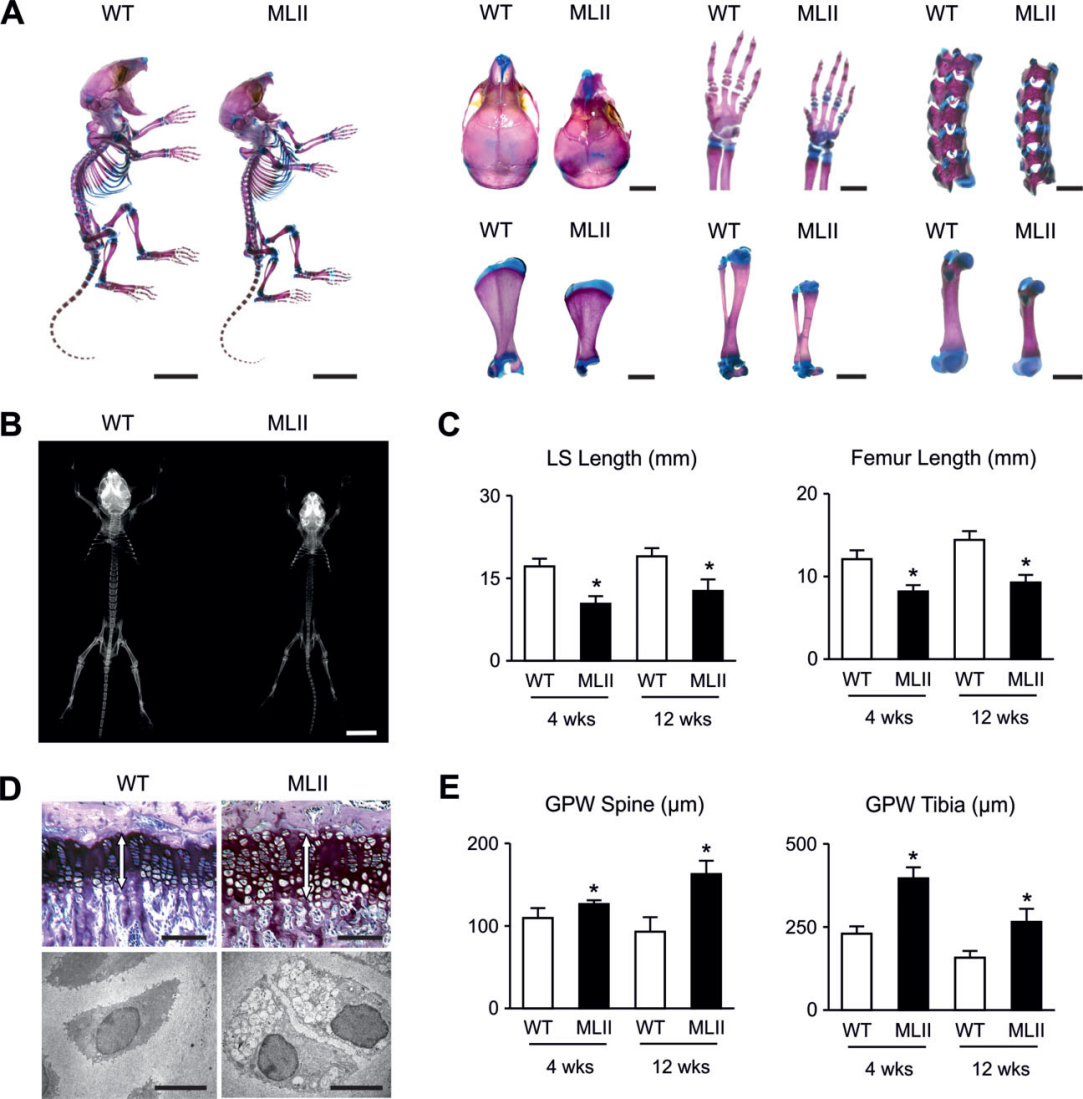
MLII mice are retarded in growth.

To analyse the skeletal phenotype of MLII mice we first stained skeletons of one week-old wild-type and MLII littermates with alcian blue and alizarin red. Here we found that all bones of MLII mice were smaller, while no defects of skeletal patterning were observed (Fig 1A). This was confirmed by contact radiography of 4- and 12-week-old mice, where we found that lumbar spine and femur length were significantly reduced in MLII mice compared to wild-type littermates (Fig 1B and C). Since skeletal growth is primarily dependent on the coordinated differentiation of growth plate chondrocytes, we examined non-decalcified spine and tibia sections from wild-type and MLII mice and observed widening of the growth plates in the latter ones (Fig 1D and E). Moreover, electron microscopy showed lucent storage vacuoles in the chondrocytes of the prehypertrophic growth plates of the MLII mice (Fig 1D).

1. Alcian blue/alizarin red staining of the whole skeleton from 7-day-old WT and MLII littermates and magnified images of crania, forelimbs, spines, scapulae, tibiae and femurs. Scale bars: 1 cm, 2 mm.
2. Contact radiographs of whole skeletons at 4 weeks of age. Scale bar: 1 cm.
3. Quantification of lumbar spine (LS) and femoral length of 4- and 12-week-old mice. Data are shown as mean and SD (*n* = 5 mice per group). *p (LS, 4 weeks) = 0.0001, *p (LS, 12 weeks) = 0.0041, *p (Fe, 4 weeks) = 0.0002, *p (Fe, 12 weeks) = 0.0003 for WT *versus* MLII by independent Student's *t*-test.
4. Toluidine blue-stained sections from tibiae of 12-week-old mice. The white arrows in each panel indicate the growth plate (top). Scale bars: 100 µm. Ultrastructural analysis of chondrocytes from tibiae of 12-week-old mice (bottom). Scale bars: 5 µm.
5. Quantification of growth plate width (GPW) from spine and tibia of 4- and 12-week-old mice. Data are shown as mean ± SD (*n* = 5 mice per group). *p (Spine, 4 weeks) = 0.0195, *p (Spine, 12 weeks) = 0.0012, *p (Tibia, 4 weeks) = 0.0001, *p (Tibia, 12 weeks) = 0.0051 for WT *versus* MLII by independent Student's *t*-test.

**Figure 2:**
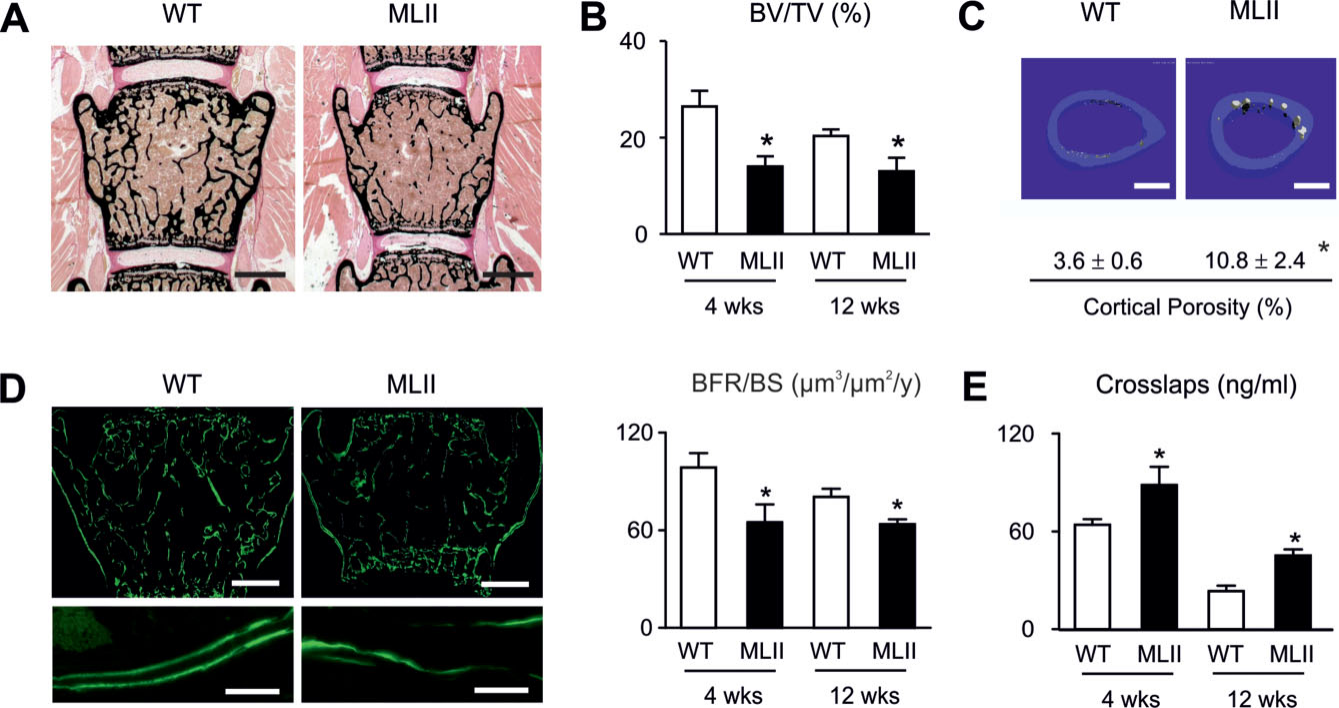
Osteopenia in MLII mice is caused by reduced bone formation and pathological increased bone resorption.

Von Kossa/van Gieson staining of the mineralized bone matrix demonstrated that the trabecular bone volume was significantly reduced in MLII mice, yet there was no pathological enrichment of non-mineralized osteoid (Fig 2A and B). Using cross-sectional microcomputed tomography (µCT) scanning of the femora we additionally observed a significant increase of cortical porosity in MLII mice, indicating enhanced bone resorption (Fig 2C). We further applied dynamic histomorphometry, a technique to determine the bone formation rate after dual injection of calcein (9 and 2 days before sacrifice), a flourescent dye binding to mineralized bone (Parfitt et al, 1987). Here we observed an overall reduction of labelled bone surfaces in MLII mice, but also reduced distances between labelling fronts, representing a lower amount of bone formation in the 7 days between the two calcein injections (Fig 2D). The quantification of these findings revealed that the bone formation rate was significantly decreased in 4- and 12-week-old MLII mice, thus suggesting that impaired osteoblast activity contributes to their low bone mass phenotype (Fig 2D). To monitor osteoclast activity we measured the serum crosslaps, representing C-telopeptide fragments of type-I-collagen being released from the bone matrix through osteoclasts and serving as a biomarker of bone resorption. We found that serum crosslaps concentrations were significantly increased in 4- and 12-week-old MLII mice, despite the fact that they display low bone mass at both ages. Taken together, these results suggested that the bone resorption rate per bone surface is more than twofold increased in MLII mice.

1. von Kossa/van Gieson staining of non-decalcified sections from vertebral bodies from 12-week-old WT and MLII mice. Scale bars: 1 mm.
2. Histomorphometric quantification of the bone volume per tissue volume (BV/TV). Data are shown as mean and SD (*n* = 5 mice per group). *p (4 weeks) = 0.0001, *p (12 weeks) = 0.0063 for WT *versus* MLII by independent Student's *t*-test.
3. Cross-sectional µCT scanning from femora and quantification of cortical porosity of 12-week-old WT and MLII mice. Scale bars: 500 µm. Values are mean and SD (*n* = 5 mice per group). *p = 0.0362 for WT *versus* MLII by independent Student's *t*-test.
4. Calcein-labelled bone surfaces of vertebral bodies from 12-week-old WT and MLII mice and their magnified image. Scale bars: 1 mm, 10 µm. Quantification of the deduced bone formation rate per bone surface (BFR/BS). Data are shown as mean and SD (*n* = 5 mice per group). *p (4 weeks) = 0.0007, *p (12 weeks) = 0.001 for WT *versus* MLII by independent Student's *t*-test.
5. Serum crosslaps in MLII and WT mice. Data are shown as mean and SD (*n* = 4 mice per group). *p (4 weeks) = 0.0041, *p (12 weeks) = 0.0001 for WT *versus* MLII by independent Student's *t*-test.

### Lysosomal storage material and increased osteoclastogenesis in MLII mice

**Figure 3:**
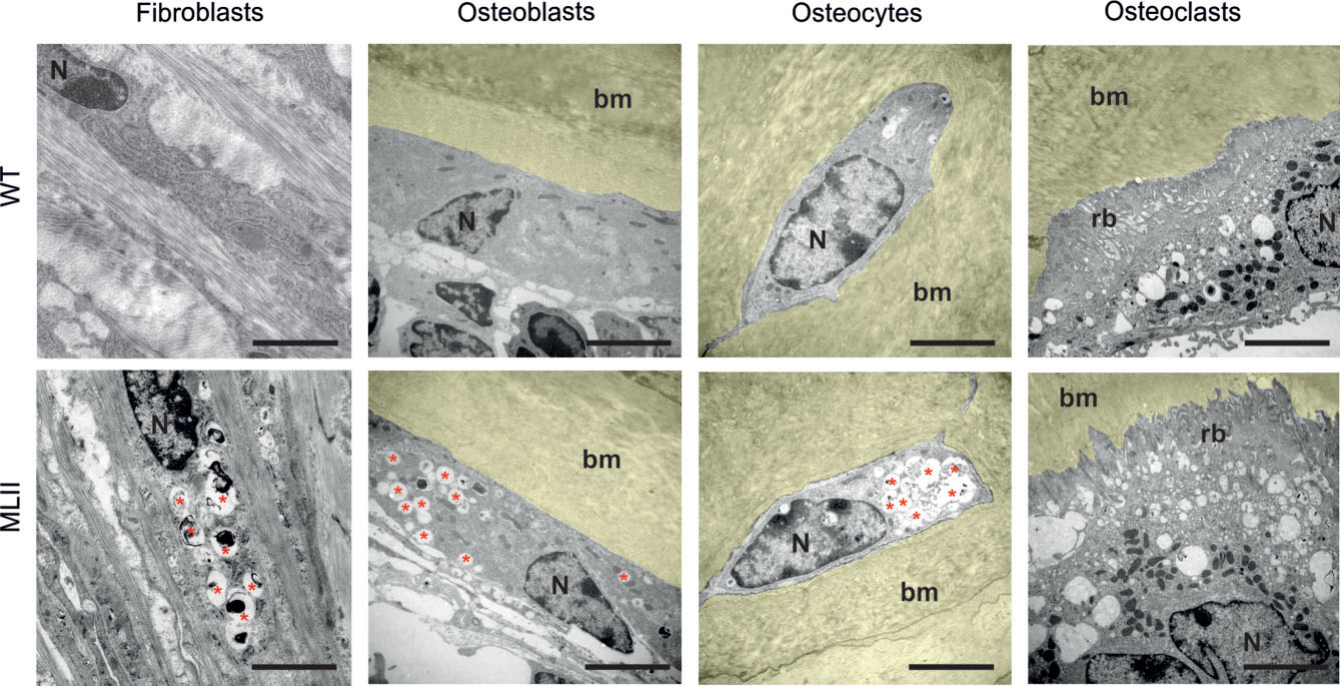
Fibroblasts, osteoblasts and osteocytes, but not osteoclasts, accumulate undegraded material in lysosomes. Ultrastructural analysis of periosteal fibroblasts, osteoblasts, osteocytes and osteoclasts from tibiae of 12-week-old MLII and WT mice (bm, bone marrow, N, nucleus, rb, ruffled border, red asterisks, storage vacuoles). Scale bars: 5 µm.

To determine whether the failure to generate the M6P targeting signal of lysosomal enzymes affects the morphology and function of bone cells, we first analysed ultra-thin bone sections from wild-type and MLII littermates by electron microscopy. Here we found lysosomal storage vacuoles characteristic for MLII in cells located in the outer fibrous layer of the periosteum, in bone-forming osteoblasts, and in terminally differentiated osteocytes (Fig 3). In contrast to these mononuclear cell types, osteoclasts are highly specialized cells with many unique morphological characteristics (Boyle et al, 2003). They are giant cells forming a ruffled border towards the bone surface, and contain many intracellular organelles, including vacuoles containing acid phosphatases (Doty & Schofield, 1972; Lucht, 1971; Schenk et al, 1967; Scott, 1967). Although their appearance in histological sections is generally more variable compared to bone-forming cell types, our systematic analysis did not reveal any morphological difference between osteoclasts from wild-type and MLII mice.

**Figure 4:**
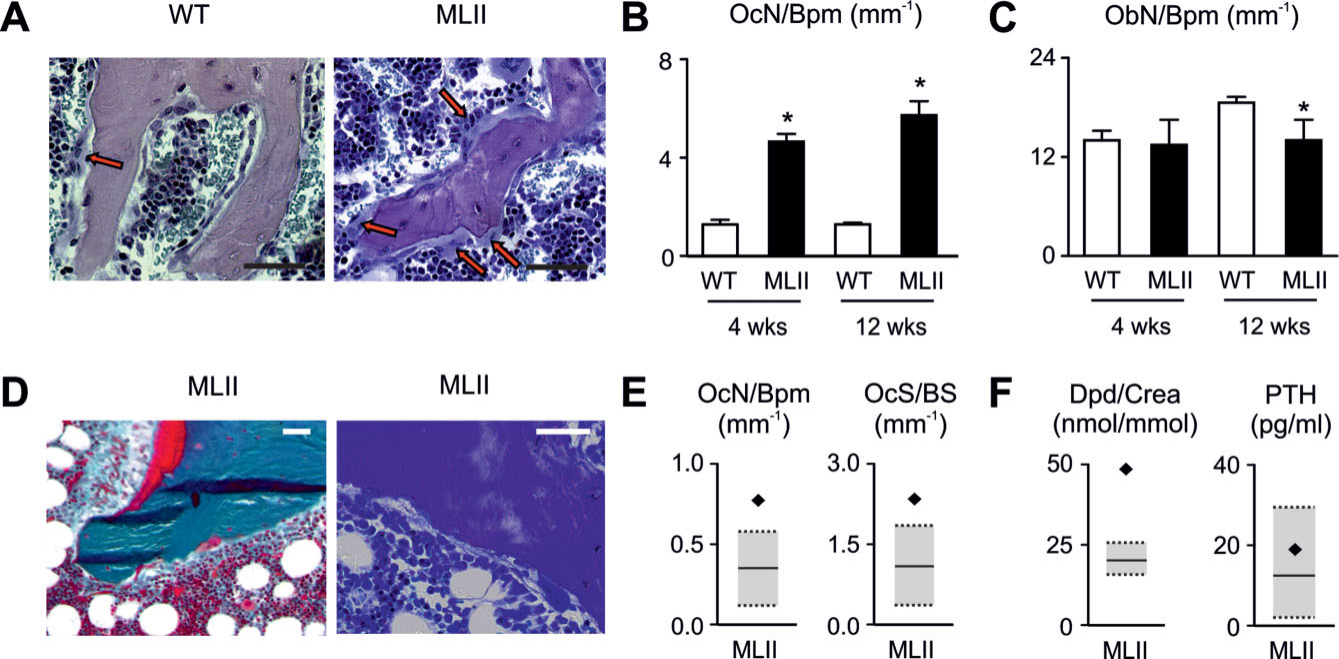
Elevated bone resorption results from pathological increased osteoclastogenesis in MLII.

We next applied cellular histomorphometry on toluidine blue-stained bone sections from 4- and 12-week-old wild-type and MLII littermates (Fig 4A). Here we found, unexpectedly, that the number of osteoclasts per bone perimeter was increased more than fourfold in MLII mice at both ages (Fig 4B). In contrast, the osteoblast number was similar between wild-type and MLII mice at the age of 4 weeks, yet slightly reduced at the age of 12 weeks (Fig 4A and C). To address the question, whether the same alteration of osteoclastogenesis is relevant for MLII in humans, we analysed a non-decalcified iliac crest biopsy from a 3-year-old female MLII patient (Fig 4D). Here, we observed a moderate enrichment of the osteoid surface, areas with fibrotic bone marrow, but also increased numbers of osteoclasts (Fig 4D and E). Since these observations represent three typical histological features of hyperparathyroidism (Parisien et al, 1990), and neonatal hyperparathyroidism has been occasionally reported in MLII patients (Unger et al, 2005), we determined serum concentrations of PTH together with the urinary concentrations of desoxypyridinoline (Dpd), a type I collagen degradation product, corresponding to crosslaps in mice (Fig 4F). In comparison to the reported children reference ranges (Cioffi et al, 2000; Husain et al, 1999) we found that the serum PTH concentration was not pathologically altered in the MLII patient. In contrast, the urinary Dpd concentration was twofold higher compared to the reference values (Fig 4F). These results demonstrate that increased bone resorption due to excessive osteclastogenesis contributes to the skeletal pathologies in MLII.

1. Toluidine blue-stained non-decalcified sections from vertebral bodies of 12-week-old WT and MLII mice. The red arrows in each panel indicate osteoclasts. Scale bars: 50 µm.
2. Quantification of osteoclast number per bone perimeter (OcN/Bpm) in vertebral bodies of 4-week-old and 12-week-old mice. Data are shown as mean and SD (*n* = 5 mice per group). *p (4 weeks) = 0.0001, *p (12 weeks) = 0.0001 for WT *versus* MLII by independent Student's *t*-test.
3. Quantification of osteoblast number per bone perimeter (ObN/Bpm) in vertebral bodies of 4-week-old and 12-week-old mice. Data are shown as mean and SD (*n* = 5 mice per group). *p (12 weeks) = 0.0228 for WT *versus* MLII by independent Student's *t*-test.
4. Goldner and toluidine staining of a non-decalcified section from a human iliac crest bone biopsy of a 3-year-old girl diagnosed with MLII. Scale bars: 100 µm.
5. Quantification of osteoclast number per bone perimeter (OcN/Bpm) and osteoclast surface per bone surface (OcS/BS) of the human MLII biopsy in relation to the age- and gender-matched reference range (Glorieux et al, 2000) (grey boxes).
6. Urinary desoxypyridinoline creatinine ratio (Dpd/Crea) and serum PTH of the 3-year-old MLII patient in relation to the age- and gender-matched reference range (Cioffi et al, 2000; Husain et al, 1999) (grey boxes).

### Missorting of lysosomal enzymes causes no functional abnormalities in MLII osteoclasts

**Figure 5:**
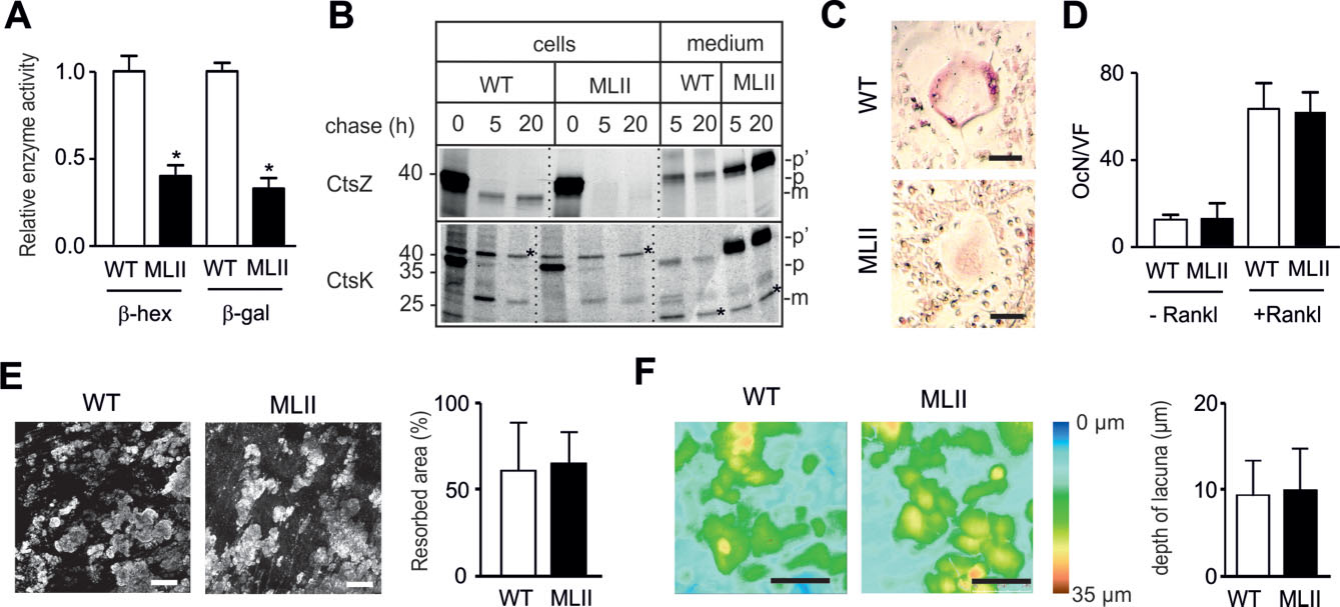
Mistargeting of lysosomal hydrolases does not result in increased resorptive capacity of MLII osteoclasts.

To determine whether the loss of M6P residues is associated with missorting of lysosomal enzymes and affects the function of osteoclasts, we isolated bone marrow cells from the femora of 12-week-old wild-type and MLII mice and differentiated them *ex vivo*. This was achieved by culturing the cells in the presence of 1,25-dihydroxy-vitamin D3 for 10 days, with soluble Rank ligand (sRankl) and M-CSF being added from day 4 until day 10 to induce osteoclastogenesis (Huebner et al, 2006). In cultured MLII osteoclasts, the specific activities of two lysosomal hydrolases, β-hexosaminidase (β-hex) and β-galactosidase (β-gal) were markedly reduced (by 60–70%) compared with controls (Fig 5A). We next performed pulse-chase experiments with [^35^S]-methionine followed by immunoprecipitation of the lysosomal proteases cathepsin Z (CtsZ) and cathepsin K (CtsK). In MLII osteoclasts the newly synthesized CtsZ was completely secreted into the medium as a precursor form during the 20-hour chase period (Fig 5B). In wild-type cells the 38 kDa [^35^S]-CtsZ precursor is proteolytically processed into 36 kDa mature forms that retained within the cells and 60% of the newly synthesized protease was secreted (Fig 5B). In contrast, CtsK which is abundantly expressed in osteoclasts was partially retained in MLII cells (Fig 5B). In MLII osteoclasts, the lack of M6P modification resulted in processing of the high mannose-type oligosaccharides and the formation of complex sugar chains on CtsZ and CtsK accompanied by the decrease of their electrophoretic mobility upon secretion (Fig 5B). In addition, we found decreased activity staining of the M6P-containing osteoclast marker protein TRAP in MLII osteoclasts (Fig 5C). These data indicate variations in the extent of M6P-dependent sorting of different lysosomal enzymes and the existence of alternative enzyme-specific M6P-independent transport routes to lysosomes.

1. Relative intracellular enzyme activity of lysosomal hydrolases β-hexosaminidase (β-hex) and β-galactosidase (β-gal) in primary cultured osteoclasts from MLII mice compared to WT. Data are shown as mean and SD (*n* = 3 primary cultures per group). *p (β-hex) = 0.0001, *p (β-gal) = 0.0001 for WT *versus* MLII by independent Student's *t*-test.
2. Primary cultures of osteoclasts from MLII and WT mice were incubated with [^35^S]-methionine for 1 h and either harvested (0 h) or chased for 5 and 20 h, followed by immunoprecipitation of lysosomal proteases CtsZ and CtsK from cell extracts and media (p, precursor posttranslational modified by high-mannose type *N*-glycans; p′, precursor modified with *N*-glycans processed to the complex type form, m, mature form).
3. Primary cultured osteoclasts from WT and MLII mice stained for TRAP activity.
4. Number of osteoclasts per visual field (OcN/VF) formed after 6 days of differentiation with 1,25(OH)_2_-vitamin D3 alone (−Rankl) (*n* = 6 primary cultures per group) or for the last 3 days in the additional presence of M-CSF and Rankl (+Rankl) (*n* = 3 primary cultures per group). Data are shown as mean and SD.
5. Quantification of area covered by resorption pits after 12-days-cultivation of osteoclasts on dentine chips. Scale bars = 50 µm. Data are shown as mean and SD (*n* = 3 primary cultures per group).
6. Topographic maps of resorption pits visualized by three dimensional stack imaging. Scale bars: 50 µm. Quantification of pit depth (WT, *n* = 275 pits; MLII, *n* = 285 pits). All values are mean and SD.

Despite the fact that osteoclasts from MLII mice could be poorly visualized by TRAP activity staining, they were quantifiable based on their unique morphology and multinuclearity. Here we found that MLII bone marrow cells did not give rise to a higher osteoclast number *ex vivo* in two experimental settings (Fig 5D). More specifically, the number of osteoclasts was not significantly different between wild-type and MLII cultures exposed to sRankl and M-CSF, as described above. The same observation was made, when the cells were only cultured in the presence of 1,25-dihydroxy-vitamin D3, a known stimulator of endogenous Rankl production in bone marrow stromal cells. While these results suggested that there is no intrinsic impairment of osteoclast differentiation, we next determined whether hypersecretion of lysosomal enzymes would affect the resorptive activity of MLII osteoclasts. For that purpose the bone marrow cells were plated on dentine slices followed by differentiation for 12 days in the presence of 1,25-dihydroxy-vitamin D3, sRankl and M-CSF. We found that neither the area of MLII resorption pits (Fig 5E) nor their depth (Fig 5F) was altered. Together, these results demonstrate that osteoclasts from MLII mice are not functionally impaired despite the hypersecretion of newly synthesized lysosomal hydrolases and that their increased differentiation capacity is not caused in a cell-autonomous manner.

### Lysosomal dysfunction and impaired differentiation of MLII osteoblasts are associated with increased expression of interleukin-6

**Figure 6:**
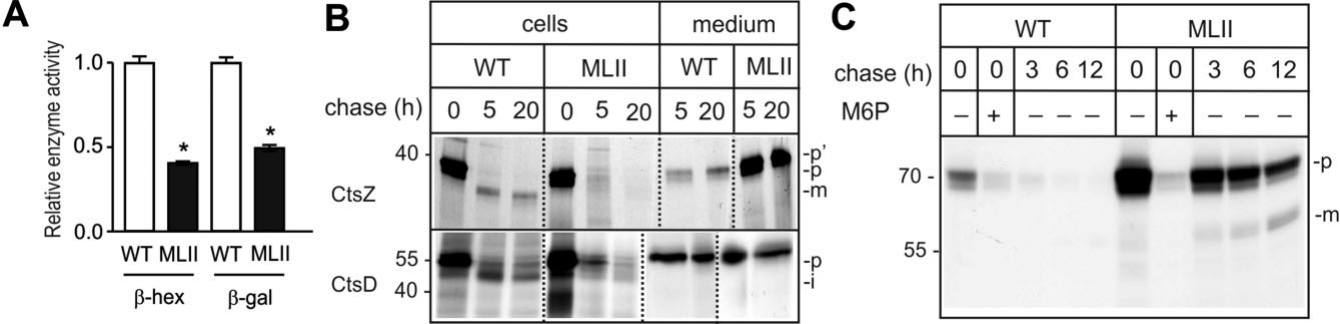
Mistargeting of lysosomal hydrolases leads to lysosomal dysfunction in MLII osteoblasts.

To analyse osteoblast differentiation and function *ex vivo*, cells were released by collagenase digestion of calvariae from 5-day-old wild-type and MLII mice and differentiated in the presence of ascorbic acid and β-glycerophosphate for 10 days (Ducy et al, 1999; Schmidt et al, 2005). Cultured MLII osteoblasts also showed reduced intracellular enzyme activities of the lysosomal enzymes β-hex and β-gal (Fig 6A). In addition, pulse-chase experiments revealed almost complete missorting of newly synthesized CtsZ precursor into the medium of MLII osteoblasts (Fig 6B) whereas the 51 kDa cathepsin D precursor (CtsD) was found partially retained in MLII osteoblasts and proteolytically processed to the 47 kDa form, suggesting its lysosomal delivery via M6P-independent pathways (Fig 6B). Incubation of wild-type and MLII osteoblasts with [^125^I]-labelled M6P-containing arylsulphatase B (ASB) showed that MLII osteoblasts internalized 3.5-fold higher amounts of [^125^I]-labelled ASB precursor in an M6P-dependent manner compared to wild-type cells suggesting that the lysosomal delivery along the endocytic pathway is affected in MLII cells. In addition, the degradation and proteolytic processing of [^125^I]-ASB was strongly delayed (Fig 6C) indicating an impairment of lysosomal functions.

1. Relative intracellular enzyme activity of lysosomal hydrolases β-hexosaminidase (β-hex) and β-galactosidase (β-gal) in primary cultured osteoblasts from MLII mice compared to WT. Data are shown as mean and SD (*n* = 3 primary cultures per group). *p (β-hex) = 0.0001, *p (β-gal) = 0.0002 for WT *versus* MLII by independent Student's *t*-test.
2. Primary cultures of osteoblasts from MLII and WT mice were incubated with [^35^S]-methionine for 1 h and either harvested (0 h) or chased for 5 and 20 h, followed by immunoprecipitation of lysosomal proteases CtsZ and CtsD from cell extracts and media (p, precursor posttranslational modified by high-mannose type *N*-glycans; p′, precursor modified with *N*-glycans processed to the complex type form, m, mature form).
3. Osteoblasts were incubated with [I^125^]-labelled arylsulphatase B (ASB) in the absence or presence of 10 mM mannose 6-phosphate (M6P). Cells were either harvested or chased for 3, 6 and 12 h. (p, precursor form; m, mature form).

**Figure 7:**
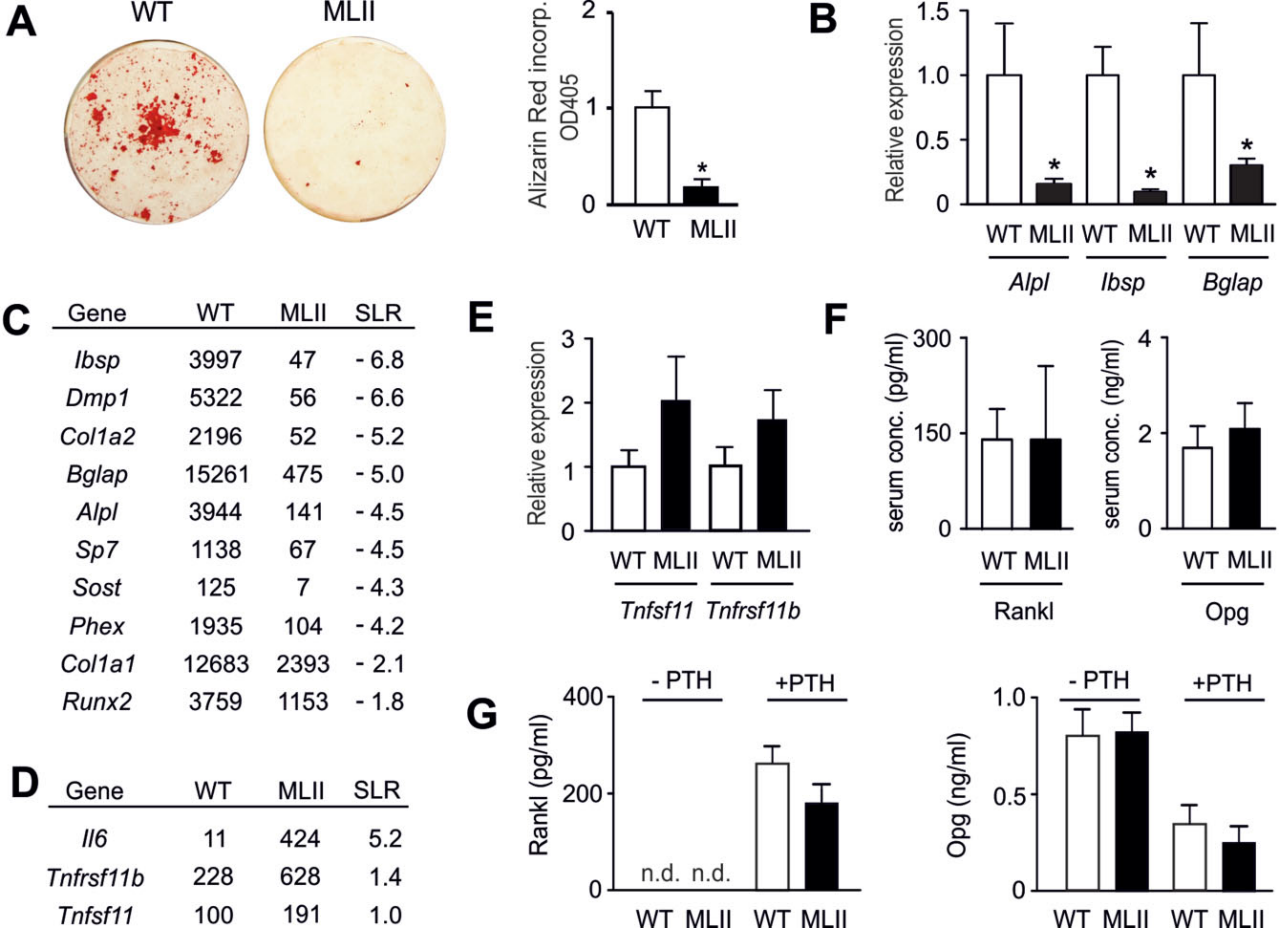
Impaired differentiation of MLII osteoblasts is not associated with altered Rankl/Opg production.

We next assessed the capability of wild-type and MLII osteoblasts to form mineralized matrix *ex vivo*. A severe reduction of alizarin red incorporation into MLII cells at day 10 of osteogenic differentiation was observed (Fig 7A). This was confirmed by qRT-PCR demonstrating that MLII cultures display reduced expression of osteoblast differentiation markers, such as *Alpl* (encoding tissue non-specific alkaline phosphatase), *Ibsp* (encoding bone sialoprotein) and *Bglap* (encoding osteocalcin) (Fig 7B). To analyse genome-wide transcriptional changes between wild-type and MLII osteoblasts we performed Gene Chip hybridization with mRNA from two independently isolated cultures of each genotype. Here we found that the most relevant genes involved in osteoblast differentiation were expressed at lower level in MLII cells, as expected (Fig 7C). With respect to genes regulating osteoclastogenesis, it was interesting that *Tnfsf11* (encoding Rankl) and *Tnfrsf11b* (encoding the Rankl antagonist Opg) were both expressed at higher levels in MLII cells. Moreover, the expression of *Il6*, encoding a pro-osteoclastogenic cytokine, was markedly increased in MLII cells (Fig 7D). To follow up on these observations we first monitored *Tnfsf11* and *Tnfrsf11b* expression in independently isolated osteoblast cultures and found a non-significant increase in MLII cells for both genes (Fig 7E). Since Rankl and Opg are also present in the circulation, we further determined their serum concentrations in 12-week-old wild-type and MLII mice and did not observe significant differences (Fig 7F). We next examined the possibility that the Rankl/Opg ratio is different between wild-type and MLII osteoblasts following stimulation with PTH, a critical regulator of bone remodelling known to affect *Tnfsf11* and *Tnfrsf11b* expression. Here we found, as expected, that short-term treatment of wild-type osteoblasts with PTH stimulated Rankl release into the medium, while the Opg concentration was reduced (Fig 7G). Most importantly however, there was no difference in the response to PTH between wild-type and MLII osteoblasts. These data suggested that the increased osteoclastogenesis observed in MLII mice is rather caused by the strong expression of Il-6 than by a change in the ratio of osteoblast-derived Rankl and Opg.

1. Alizarin red staining of primary osteoblast cultures. Quantification of Alizarin Red incorporation. Data are shown as mean and SD (*n* = 3 primary cultures per group). *p = 0.0003 for WT *versus* MLII by independent Student's *t*-test.
2. qRT-PCR expression analysis for the indicated osteoblastogenesis markers at day 10 of differentiation. Data are shown as mean and SD (*n* = 3 cultures per group). *p (*Alpl*) = 0.0217, *p (*Ibsp*) = 0.0020, *p (*Bglap*) = 0.0360 for WT *versus* MLII by independent Student's *t*-test.
3. Expression of osteoblastogenesis markers assessed by Gene Chip hybridization. Shown are the mean signal intensities and logarithmic signal ratios (SLR, signal log ratio) between wild-type and MLII samples (combined from two independent experiments).
4. Expression of osteoclastogenesis regulators assessed by Gene Chip hybridization. Shown are the mean signal intensities and logarithmic signal ratios (SLR, signal log ratio) between wild-type and MLII samples (combined from two independent experiments).
5. qRT-PCR expression analysis for the indicated osteoclastogenesis regulators at day 10 of differentiation. Data are shown as mean and SD (*n* = 3 cultures per group).
6. Serum concentration of Rankl and Opg in 12 week-old mice. Data are shown as mean and SD (*n* = 5 mice per group).
7. Concentration of Rankl and Opg in conditioned medium of cultured primary osteoblasts with or without PTH treatment as indicated. Data are shown as mean and SD (*n* = 3 primary cultures per group).

**Figure 8:**
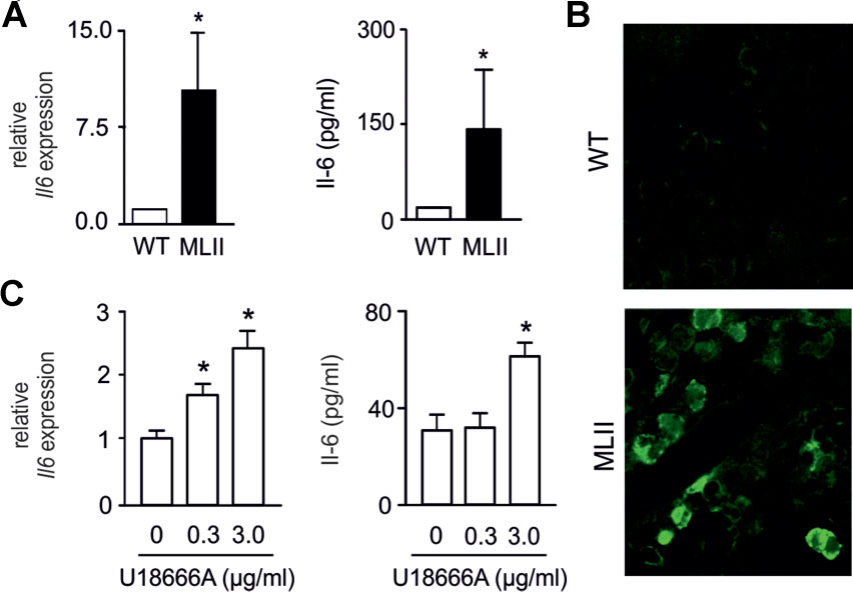
Impaired differentiation of MLII osteoblasts is associated with increased interleukin-6 production.

Although Il-6 is primarily known for its pro-inflammatory role in the immune system, it also acts as a potent stimulator of osteoclastogenesis *in vitro* and *in vivo* (De Benedetti et al, 2006; Ishimi et al, 1990; Kudo et al, 2003). By qRT-PCR we found that *Il6* expression was more than fivefold increased in MLII osteoblasts (Fig 8A). Likewise, Il-6 concentrations were markedly increased in the medium of MLII osteoblasts (Fig 8A). Finally, Il-6 immunoreactivity was specifically detectable in bone marrow cells in sections of the tibia of MLII mice (Fig 8B). To examine whether storage material, such as unesterified cholesterol accumulating in several tissues of MLII mice (Kollmann et al, 2012), can induce *Il6* expression, cultured wild-type osteoblasts were incubated with U18666A, a hydrophobic amine leading to the accumulation of cholesterol in late endosomes/lysosomes (Cenedella, 2009). Here we found that U18666A treatment significantly increased *Il6* expression and Il-6 protein concentrations in the medium of osteoblasts in a concentration-dependent manner (Fig 8C). These studies identified Il-6 as one candidate, whose elevated expression in MLII osteoblasts contributes to increased osteoclastogenesis in MLII mice.

1. qRT-PCR expression analysis for *Il6* on primary osteoblast cultures and enzyme immunoassay-based determination of Il-6 concentrations in medium of cultured primary osteoblasts. Data are shown as mean and SD. *p (RT-PCR) = 0.0124 for WT *versus* MLII (*n* = 3 primary cultures per group). Relative mRNA expression, normalized to the level of *Gapdh* mRNA, was calculated using the comparative CT method (2^–ΔΔC(T)^). *p (ELISA) = 0.0455 for WT *versus* MLII by independent Student's *t*-test.
2. Immuno-histological staining for Il-6 in bone marrow cells in sections of tibia from 12 week-old mice.
3. To induce lysosomal storage of cholesterol primary cultured wild-type osteoblasts were incubated with 0, 0.3 and 3.0 µg/ml U18666A for 6 h 10 days after seeding. *Il6* mRNA expression was analysed by RT-PCR and Il-6 concentrations in medium of cultured primary osteoblasts were determined by enzyme immunoassay. Data are shown as mean and SD (*n* = 3 cultures per group). *p (RT-PCR) = 0.0041 for 0 *versus* 0.3 µg/ml U18666A, *p (RT-PCR) = 0.0013 for 0 *versus* 3.0 µg/ml U18666A and *p (ELISA) = 0.0004 for 0 *versus* 3.0 µg/ml U18666A by independent Student's *t*-test.

**Figure 9:**
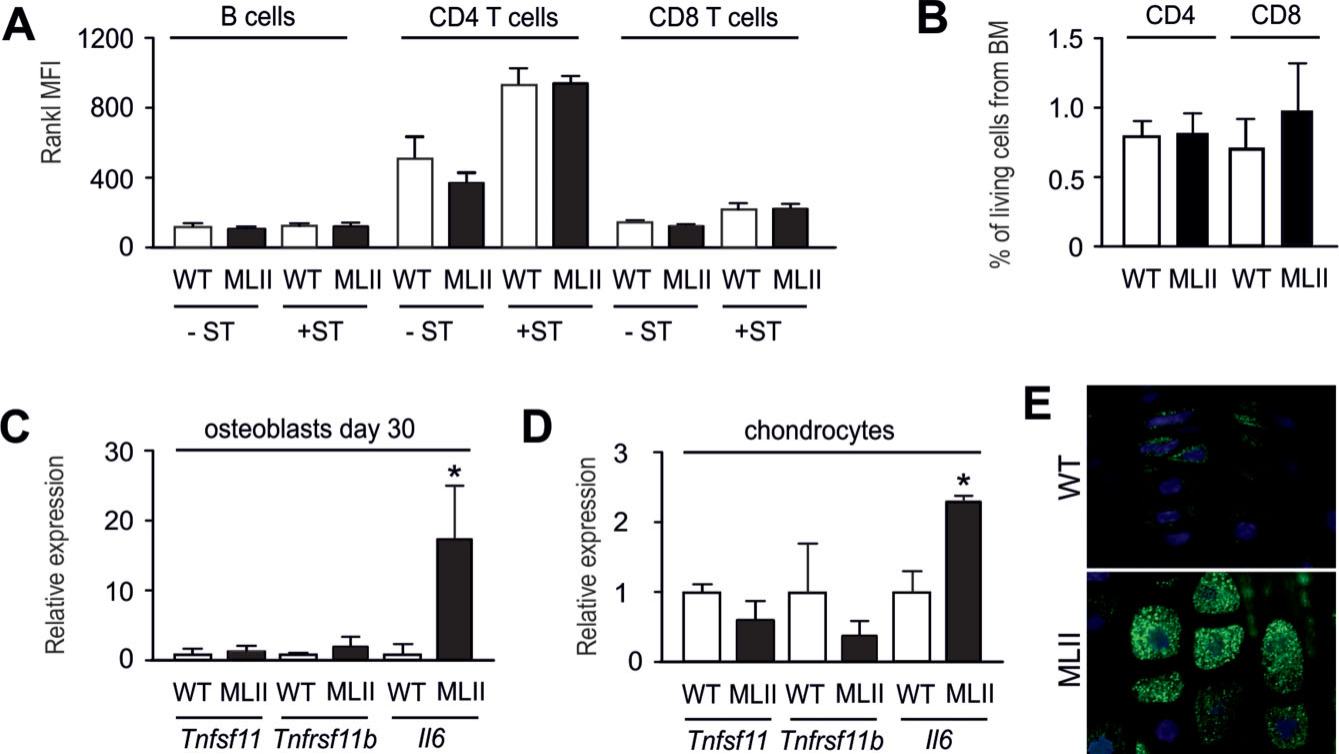
*Tnfsf11* expression is not induced in different MLII cell types.

Although it is well-established that osteoblasts are critical regulators of osteoclastogenesis, there is increasing evidence that other cell types (such as lymphocytes, osteocytes or chondrocytes) are a significant source of Rankl thereby controlling bone resorption (Kong et al, 1999; Nakashima et al, 2011; Xiong et al, 2011). Therefore, it was important to analyse the possibility that Rankl production is increased in MLII cell types other than osteoblasts. For that purpose we first isolated lymphocytes (B cells, CD4^+^ and CD8^+^ T cells) from the spleen of 12 week-old wild-type and MLII mice and quantified the expression of Rankl protein by FACS analysis. We found highest Rankl expression in CD4^+^ T cells upon stimulation with PMA and ionomycin, but that there was no significant difference between the wild-type and MLII lymphocyte (Fig 9A). Since we observed no alterations in the number of CD4^+^ and CD8^+^ T cells in the bone marrow of 12 week-old MLII mice either (Fig 9B), it appears unlikely that increased Rankl production by T cells is causing the increased osteoclastogenesis in MLII mice. We next analysed the expression of *Tnfsf11*, *Tnfrsf11b* and *Il6* in terminally differentiated osteoblasts (day 30 of differentiation) and in primary chondrocytes (day 20 of differentiation). Here we found that neither Rankl- nor Opg-encoding genes were differentially expressed in wild-type and MLII cultures (Fig 9C and D). In contrast, there was a more than 10-fold induction of *Il6* expression in terminally differentiated osteoblasts from MLII mice, and the *Il6* mRNA level was significantly increased in MLII chondrocytes. Immunohistochemistry further demonstrated that hypertrophic chondrocytes of MLII mice are Il-6-positive in comparison to wild-type chondrocytes (Fig 9E). Taken together, these findings suggested that the skeletal phenotype of MLII mice is not caused by changes in Rankl production, but that ectopic production of Il-6 may cause a Rankl-independent induction of osteoclastogenesis.

1. Determination of the mean fluorescence intensity (MFI) of the Rankl signal in B cell, CD4 T cell and CD8 T cell populations isolated from spleen (-ST). Prior to the FACS analysis one group of isolated cells were additionally stimulated with PMA and ionomycin for 4 h (+ST) to induce an immune response. Data are shown as mean + SD (*n* = 3 cell preparations per group).
2. Quantification of CD4 T cells and CD8 T cells in living cell population isolated from bone marrow using FACS. Data are shown as mean + SD (*n* = 3 mice per group).
3. qRT-PCR expression analysis for the indicated osteoclastogenesis regulators in terminally differentiated osteoblasts (day 30 of differentiation). Data are shown as mean and SD (*n* = 3 cultures per group). *p (*Il6*) = 0.0360 for WT *versus* MLII by independent Student's *t*-test.
4. qRT-PCR expression analysis for the indicated osteoclastogenesis regulators in primary chondrocytes (day 20 of differentiation). Data are shown as mean and SD (*n* = 3 cultures per group). *p (*Il6*) = 0.0041 for WT *versus* MLII by independent Student's *t*-test.
5. Immunohistochemical staining of Il-6 in growth plate chondrocytes from 12 week-old mice.

### Bisphosphonates increase bone mass and stability in MLII mice

**Figure 10:**
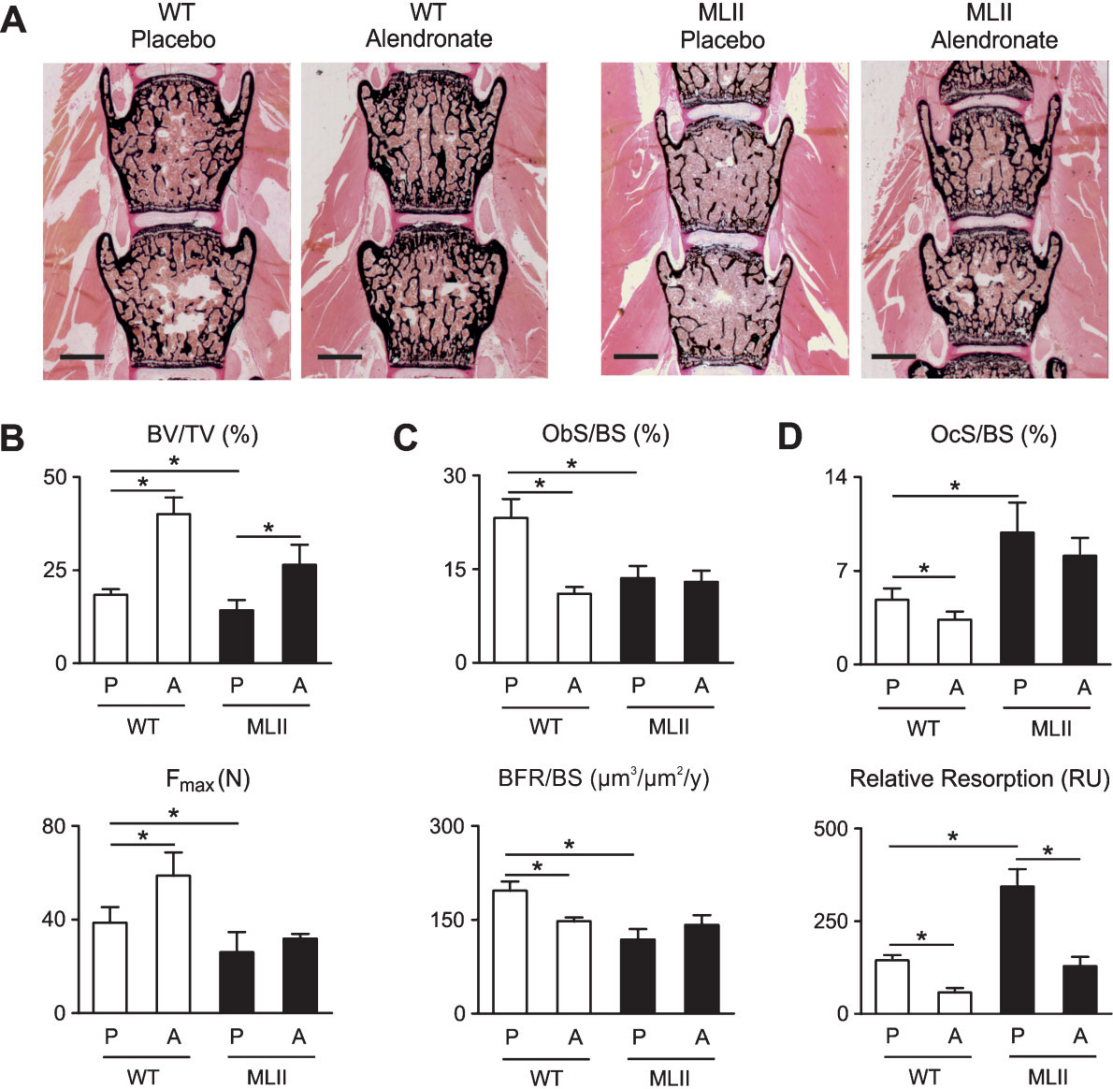
The osteopenia in MLII mice can be prevented by bisphosphonate treatment.

Given the striking increase of osteoclastogenesis in MLII mice, we finally addressed the question, of whether their low bone mass phenotype can be corrected by alendronate, a potent anti-resorptive bisphosphonate, negatively affecting osteoclasts (Russell, 2011). For that purpose we treated wild-type and MLII mice by semiweekly injections of alendronate for 8 weeks and examined the non-decalcified bones thereafter (Fig 10A). The trabecular bone volume was significantly increased by alendronate administration in both wild-type and MLII mice, and microcompression studies showed that the reduced biomechanical stability of ML II vertebral bodies was normalized by alendronate treatment (Fig 10B). Cellular and dynamic histomorphometry further revealed that alendronate treatment of wild-type mice caused the expected reduction of osteoblast surface and bone formation rate, possibly explained by the fact that osteoclasts produce osteoanabolic factors (Henriksen et al, 2013). In the case of MLII both parameters were significantly reduced in the placebo group, but there was no further reduction of osteoblast surface and bone formation rate by alendronate treatment (Fig 10C). A similar finding was made for the osteoclast surface, which was increased in MLII mice, but not affected by alendronate. In contrast, the osteoclast surface was significantly reduced by alendronate treatment of wild-type mice (Fig 10D). Most importantly however, when we normalized the serum crosslaps concentrations to the bone mass, we observed that alendronate treatment significantly reduced the rate of bone resorption in both genotypes, as expected.

1. von Kossa/van Gieson staining of non-decalcified sections from vertebral bodies of 12-week-old WT and MLII mice after semiweekly injections with placebo (P) or alendronate (A) for 8 weeks. Scale bars: 1 mm.
2. Quantification of the trabecular bone volume per tissue volume (BV/TV) and the force to failure (*F*_max_) in microcompression testing of vertebral bodies. Values are mean and SD (*n* = 5 mice per group). *p (BV/TV) = 0.0218 for WT (P) *versus* MLII (P), *p (BV/TV) = 0.0001 for WT (P) *versus* WT (A), *p (BV/TV) = 0.0045 for MLII (P) *versus* MLII (A), *p (*F*_max_) = 0.0105 for WT (P) *versus* MLII (P), *p (*F*_max_) = 0.0465 for WT (P) *versus* WT (A) by independent Student's *t*-test.
3. Quantification of osteoblast surface per bone surface (ObS/BS) and the bone formation rate per bone surface (BFR/BS). Values are mean and SD (*n* = 5 mice per group). *p (ObS/BS) = 0.0009 for WT (P) *versus* MLII (P), *p (ObS/BS) = 0.0001 for WT (P) *versus* WT (A), *p (BFR/BS) = 0.0004 for WT (P) *versus* MLII (P), *p (BFR/BS) = 0.0002 for WT (P) *versus* WT (A) by independent Student's *t*-test.
4. Quantification of osteoclast surface per bone surface (OcS/BS) and the relative bone resorption (serum crosslaps concentration normalized to bone mass; RU, relative units). Values are mean and SD (*n* = 5 mice per group). *p (OcS/BS) = 0.0023 for WT (P) *versus* MLII (P), *p (OcS/BS) = 0.0136 for WT (P) *versus* WT (A), *p (Res) = 0.0001 for WT (P) *versus* MLII (P), *p (Res) = 0.0001 for WT (P) *versus* WT (A), *p (Res) = 0.0005 for MLII (P) *versus* MLII (A) by independent Student's *t*-test.

## DISCUSSION

The skeletal phenotype observed in MLII mice is highly similar to that in humans: short vertebral bodies, widened diaphyses and generalized bone loss (Braulke et al, 2013; Cathey et al, 2010; David-Vizcarra et al, 2010; Robinson et al, 2002; Spranger & Wiedemann, 1970). Our biochemical findings demonstrate increased bone resorption with bone-specific collagen degradation products (crosslaps) in the serum of MLII mice. In addition, our studies show that the impaired bone formation rate is most likely explained by accumulation of storage material in osteoblasts. Most importantly, the MLII mouse data and the analysis of a human iliac crest biopsy sample demonstrate that increased osteoclastogenesis is a major skeletal pathomechanism in MLII. These unexpected findings underscore the relevance of a deep systematic phenotypic analysis assessing skeletal remodelling, which should also be applied for other lysosomal storage disorders, whose accompanying skeletal defects are poorly understood.

In addition, our data reveal that osteoclasts, unlike chondrocytes and osteoblasts, are functionally not affected by missorting of lysosomal enzymes lacking M6P residues, which is believed to be responsible for increased bone resorption activity of MLII osteoclasts (van Meel et al, 2011). Our findings, however, suggest that in MLII osteoclasts the polarized sorting of lysosomal enzymes towards the apical resorption lacunae (Baron et al, 1988) is disturbed and result in a non-directed transport to basolateral membranes without affecting resorbing activity of osteoclasts. Furthermore, a variable, cell-type- and tissue-specific targeting efficiency of lysosomal enzymes has been reported in human and mouse MLII (Boonen et al, 2011; Glickman & Kornfeld, 1993; Kollmann et al, 2012; Owada & Neufeld, 1982; van Meel et al, 2011). Here we show that the lack of M6P residues in MLII osteoblasts and osteoclasts led to loss and missorting of cathepsin Z whereas β-hexosaminidase, β-galactosidase, cathepsin K and cathepsin D can partly reach lysosomes in an M6P-independent manner. Alternative receptor proteins with compensatory and selective properties for lysosomal enzymes and/or cell-specific substrates might be responsible for cellular differences in lysosomal functionality and subsequent accumulation of lysosomal storage material.

It is evident that the transcription of several genes, including the proinflammatory cytokine Il-6, is upregulated in dysfunctional MLII osteoblasts or in osteoblasts from wild-type mice showing U18666A-induced accumulation of unesterified cholesterol (Cenedella, 2009). Although unesterified cholesterol was also found in fibroblasts and brain tissue of MLII mice (Kollmann et al, 2012) the increased level of Il-6 appears to be not specific for MLII and has been reported in fibroblasts and brain tissue of mice with the lysosomal cholesterol-storage disorder Niemann-Pick type C (Suzuki et al, 2007). It has been shown that Il-6 activates the expression of the major pro-osteoclastogenic cytokine Rankl (Ishimi et al, 1990; Kudo et al, 2003; Teitelbaum & Ross, 2003). Since the expression and release of Rankl is not altered in MLII osteoblasts, it is likely that increased levels of Il-6 induce osteoclastogenesis in MLII mice by Rankl-independent mechanisms (Kudo et al, 2003) in agreement with pro-osteoclastogenic functions of Il-6 in transgenic mice which display osteopenia and increased osteoclastogenesis (De Benedetti et al, 2006), and Il-6-deficient mice which are protected from ovariectomy-induced bone loss (Poli et al, 1994).

Skeletal radiographs of newborn MLII patients resemble those seen in neonatal hyperparathyroidism (Parisien et al, 1990). In the MLII mice and the MLII patient described here, we did not find increased serum PTH concentrations which is in agreement with reports on more than 25 MLII patients with normal PTH and parathyroid receptor protein concentrations (David-Vizcarra et al, 2010; Otomo et al, 2011). Only some cases of MLII are associated with marked elevation of serum PTH levels (Unger et al, 2005). Our data also exclude tissue hypersensitivity (pseudohyperparathyroidism) to PTH postulated as pathogenetic mechanism for the osteopenia (David-Vizcarra et al, 2010; Parisien et al, 1990), since the secretion of Rankl in osteoblasts from MLII mice was not affected in response to PTH stimulation.

Most importantly, we were able to demonstrate that increased osteoclastogenesis is essentially the first pathologic symptom in MLII that is treatable by established medications, thereby raising the question, if affected individuals would profit from anti-resorptive therapy. Bisphosphonate administration, however, is usually not applied for growing children, unless they have a high fracture risk, for instance in the case of osteogenesis imperfecta (Silverman, 2011). Based on the severity of the other organ pathologies in MLII it is therefore debatable, if a similar strategy should be applied here. This is further complicated by the rarity of the disease that essentially rules out the application of clinical studies to define standard treatment regimes. There are, however, case reports where individuals with MLII or the related disease MLIII have profited from bisphosphonate treatment (Robinson et al, 2002). In addition, bisphosphonates have been successfully applied for a subset of individuals with Gaucher disease respresenting the most prevalent lysosomal storage disorder (Cox et al, 2008). This implies that understanding the precise pathomechanisms underlying the skeletal defects in this large group of inherited disorder is of importance, and that a profound histomorphometric analysis should be performed on bone sections from mouse models and/or patients with defined lysosomal storage disorders.

## MATERIALS AND METHODS

### Animals

The MLII mouse model, generated by the insertion of a cytosine in the murine *Gnptab* gene (c.3082insC) that is homologous to a homozygous mutation in an MLII patient (Tiede et al, 2005), has been described previously (Kollmann et al, 2012). Experiments were performed with female MLII mice and wild-type littermates from heterozygous breedings in a mixed C57Bl/6-129/SvJ genetic background. The bone phenotypes were analysed at the ages of 1, 4 and 12 weeks. For the determination of the bone formation rate mice were given two injections of calcein (25 mg/kg body weight) 9 and 2 days before sacrifice. For treatment of bone loss, Alendronate (Sigma–Aldrich) was given in 0.9% saline solution in a dose of 150 µg/kg body weight semiweekly. Beginning at 4 weeks of age and for 8 weeks thereafter, the animals were given intraperitoneal injections of alendronate or vehicle alone, depending on their experimental group. Mice were fed a standard rodent diet and housed in a pathogen-free animal facility at the University Medical Center Hamburg-Eppendorf, and experimental procedures were performed according the institutional guidelines and approved by the Amt für Gesundheit und Verbraucherschutz.

### Human bone biopsy specimen

With informed consent of the parents a transiliac bone biopsy specimen was taken from a 3-year-old girl, diagnosed for MLII (*GNPTAB* c.344_345delCA/c.1022delC) and analysed at the Institute of Pathology and the Institute for Osteology and Biomechanics at University Medical Center Hamburg-Eppendorf. The biopsy specimen was embedded non-decalcified into methylmethacrylate. Sections were subjected to Goldner and toluidine blue staining, and histomorphometric analysis was carried out using the OsteoMeasure system (Osteometrics) following the guidelines of the American Society for Bone and Mineral Research (Parfitt et al, 1987) and compared to the age- and gender-matched reference range (Glorieux et al, 2000). The bone resorption parameter DPD/creatinine from urine and the serum PTH concentration of the patient were measured in the Department of Clinical Chemistry of the University Medical Center Hamburg Eppendorf according to standard procedures and compared to the age- and gender-matched reference range (Cioffi et al, 2000; Husain et al, 1999).

### Skeletal analysis

Alcian blue and alizarin red double-staining of whole mice at an age of 1 and 4 weeks was performed using standard protocols (McLeod, 1980). The skeletal analysis, including contact X-ray, µCT scanning and non-decalcified bone histology, has been described previously (Schmidt et al, 2005). Sections were subjected to von Kossa/van Gieson and toluidine blue staining, and histomorphometric analysis was carried out using the OsteoMeasure system (Osteometrics) (Amling et al, 1999; Parfitt et al, 1987). Nonstained sections were used to determine the bone formation rate by fluorochrome measurements. Lumbar spine, femoral length and growth-plate thickness were determined with a microscopic ruler. Microcompression assays were performed with the lumbar vertebral bodies L6 using a Z2.5/TN1S-device (Zwick). Ultrastructural analyses and immunohistochemistry was performed according to standard protocols (Weinert et al, 2010) using antibodies against mouse interleukin 6 (ab6672; Abcam).

### Primary culture of osteoclasts and functional analysis

To analyse cultured osteoclasts, bone marrow cells were isolated from the femora by flushing with α-MEM containing 10% FBS and seeded into 24-well plates or dentin chips. Marrow cultures were incubated with 10 nM 1.25(OH)_2_ vitamin D3 (Sigma), 40 ng/ml sRankl (Peprotech) and 20 ng/ml M-CSF (Peprotech) for 10 days. Medium was changed every other day (Huebner et al, 2006). Formation of multinuclear cells was assessed by TRAP activity staining as described previously (Huebner et al, 2006).

To determine the resorption activity of primary osteoclasts, 1.5 × 10^5^ cells isolated from bone marrow were differentiated on dentine chips in a 96-well dish. After 12 days of differentiation osteoclasts were lysed with PBS containing 1% SDS and removed from the dentine by sonication in PBS. The dentine slices were stained with 0.01 mg/ml biotinylated wheat-germ agglutinin (Vector lab) in PBS containing 1% bovine serum albumin for 1 h at room temperature followed by an 1 h incubation with Alexa Fluor® 546 streptavidin (Molecular Probes). The resorption pits were visualized using a confocal laser scanning microscope Leica TCS SP5 II AOBS equipped with a Diode-pumped solid-state laser emitting at 561 nm and an HCX PLAPO 63x/NA 1.40 objective. The detected fluorescence signal was collected by a new generation GaAsP Hybrid detector. The z step size was fixed to be 250 nm allowing accurate depth measurements. Three dimensional chip overviews were measured by the Leica TCS SP5 in tile scanning mode using the same laser source but an HCPL FLUOTAR 10x/NA 0.30 objective. The total area *A* of the chips and the area occupied by lacunas *A*_l_ were measured using the Velocity software and used for calculation of the free area *A*_f_ (*A*_f_ = 1 − *A*_l_/*A*).

### Primary culture of osteoblasts and chondrocytes

Cultures of primary osteoclasts and osteoblasts were generated as described previously (Huebner et al, 2006; Schmidt et al, 2005). In brief, osteoblast progenitors were isolated from individual calvariae of 5-day-old wild-type and MLII littermates. Cells were released by collagenase/dispase digestion and plated in α-MEM/10% FCS at an initial density of 10.000 cells/cm^2^. Osteoblast differentiation was induced at 80% confluency by the addition of ascorbic acid (50 µg/ml) and β-glycerophosphate (10 mM) for 10 days of culture. In one experiment the differentiation was continued until day 30 to induce an osteocyte-like state of terminal differentiation. For the assessment of *ex vivo* mineralization, primary osteoblasts were stained by alizarin red S at day 10 of differentiation induced by ascorbic acid and β-glycerophosphate.

Alizarin red incorporation was quantified by measuring the absorbance of alizarin red at 405 nm in cell lysates. To analyse the PTH sensitivity, primary osteoblasts differentiated for 10 days were incubated with 10 nM human PTH (Bachem) for 6 h and induction of Rankl mRNA expression and secretion were determined. To investigate the effect of endosomal/lysosomal storage of unesterified cholesterol on *Il6* mRNA expression and secretion, primary wild-type osteoblasts were cultured in the presence of 0.3 or 3.0 µg/ml U18666A (Sigma) for 6 h.

Chondrocyte progenitor cells were isolated from single sternums out of 12 days old wild-type and MLII littermates. Cells were separated by digesting the tissue initially in 0.1% collagenase solution followed by 0.2% collagenase solution and plated in DMEM/Ham's F-12 (1:1)/10% FCS (Biochrome AG) at an initial density of 250.000 cells/cm^2^. At a total cell confluence of 80%, chondrocyte differentiation was induced by the addition of ascorbic acid (50 µg/ml) and maintained throughout the experiment. At day 20 RNA was isolated (RNA prurification kit by Machery-Nagel) to perform real-time analysis.

### Biochemical analysis

The activity of lysosomal enzymes β-hexosaminidase and β-galactosidase in cell lysates and media from osteoblasts and osteoclasts was determined by estimation of *p*-nitrophenol liberated from the hydrolase-specific monosaccharide substrate as described previously (Marschner et al, 2011). To quantify osteoclastic bone resorption, we determined the serum concentration of type I collagen fragments with the RatLaps EIA (AC-06F1; IDS). Concentrations of hormones in serum and cell culture supernatants of primary cultured osteoblasts were quantified using antibody-based detection kits (Mouse Il-6, KMC0061; invitrogen; Rankl; MTR00, R&D Systems, Opg, 459-MO-100, R&D Systems).

### Expression analysis

Total RNA was isolated from primary cultured osteoblasts and osteoclasts using GeneJET™ RNA Purification Kit (Fermentas). For genome-wide expression analysis, procedures for cDNA synthesis, labelling and hybridization were carried out according to the manufacturer's protocol (Affymetrix). The experiment was performed using Affymetrix Mouse genome 430 2.0 Gene Chips. First strand cDNA synthesis with 100 ng of total RNA, synthesis of biotin-labelled cRNA and clean-up was carried out using the 3′ IVT Express Kit (Affymetrix). For hybridization, 15 µg of fragmented cRNA were incubated with the chip in 200 µl of hybridization solution in Hybridization Oven 640 (Affymetrix) at 45°C for 16 h. GeneChips were then washed and stained with the Affymetrix Fluidics Station 450 according to the GeneChip Expression Wash, Stain and Scan Manual using the GeneChip Hybridization, Wash and Stain Kit (Affymetrix). Microarrays were scanned with the Affymetrix GeneChip Scanner 7G, and the signals were processed using GCOS (v.1.4; Affymetrix). The GCOS software was used to scale the samples to a target signal of 200. We performed 4 arrays (*n* = 2 individual cultures of wild-type and mutant cells) and analysed the data using pairwise cross-comparison between the groups. We filtered out transcripts with an absent-call in both groups and transcripts that did not show the same regulation tendency in all comparisons (‘increase/decrease call’ = 4 of 4 total comparisons). The data have been deposited in NCBI's Gene Expression Omnibus (Edgar et al, 2002) and are accessible through GEO Series accession number GSE43854 (http://www.ncbi.nlm.nih.gov/geo/query/acc.cgi?acc=GSE43854).

For expression analysis of selected genes the Cloned AMV First-Strand cDNA Synthesis Kit (Invitrogen) was used for cDNA synthesis and TaqMan™ Gene Expression Assays (Applied Biosystems) including predesigned probes and primer sets were used for real-time analysis. Relative mRNA expression, normalized to the level of *Actb* mRNA, was calculated using the comparative CT method (2^−ΔΔC(T)^).

### Immune cell preparation and FACS analysis

Immune cells were isolated from spleen by passing through 200 µm metal sieve and 70 µm plastic cell strainer into PBS. Cells from bone marrow were isolated from the femora by flushing with PBS and passed through 70 µm plastic cell strainer. Red blood cells were lysed with lysing buffer (155 mM NH_4_Cl, 10 mM KHCO_3_, 100 µM EDTA, pH 7.2). Stimulation was performed with 50 ng/ml phorbol 12-myristate 13-acetate (PMA, Sigma) and 1 µM ionomycin (Sigma) in the presence of 10 µg/ml brefeldin A (Sigma) in complete medium (RPMI 1640 +5% FCS +1% glutamin +0.1% gentamicin +0.1% 2-mercaptoethanol) for 4 h at 37°C.

For FACS analysis cells were first stained with fluorochrome-conjugated mAb for the surface markers: antiCD4 clone RM4-5 (BD Biosciences), antiCD19 clone 1D3 (eBioscience), and antiCD8a clone 53-6.7 (eBioscience). Dead cells are stained with pacific orange succinidyl ester (Invitrogen). Staining was performed for 20 min on ice. After that cells were fixed with 2% paraformaldehyde for 20 min at room temperature. Rankl was stained with fluorochrome-conjugated mAb: antiCD254 clone IK22/5 (Biolegend) for 20 min at room temperature in permeabilization buffer (PBS +0.3% saponin +0.1% BSA). Cells were measured on a Canto II flow cytometer (BD Biosciences) and data were analysed with the FACSDiva software (BD Biosciences). Debris, doublets, and dead cells were excluded from analysis.

### Other methods

Pulse-chase experiments with [^35^S]-methionine followed by immunoprecipitation of the lysosomal proteases cathepsin Z, K and D from cell extracts and media of osteoblasts and osteoclasts were performed at day 10 of differentiation using antibodies from R&D Systems (cathepsin Z), Millipore (cathepsin K), and against purified mouse cathepsin D (Claussen et al, 1997), as described previously (Marschner et al, 2011). Recombinant human ASB kindly provided by Dr. M. Vellard (BIOMARIN, Novato, CA, USA) was iodinated with sodium I (74 TBq/mmol; Hartmann Analytic (Braunschweig, Germany) and IODO-GEN® (Pierce, Rockford, IL, USA) as described (Braulke et al, 1987). Osteoblasts grown in 35 mm dishes were incubated with [I^125^] labelled ASB in the absence or presence of 10 mM M6P (Sigma–Aldrich) for 30 min at 37°C in DMEM, 0.1% BSA. After removal of radioactive [^125^I]-ASB containing medium, cells were either harvested or chased for 3, 6 and 12 h in DMEM, 0.1% BSA. After replacement of cell surface bound [^125^I]-ASB with two washes ice-cold PBS containing 10 mM M6P for each 5 min, cells were lysed and total protein homogenates were separated by SDS-PAGE followed by autoradiography.

### Statistics

Results are presented as bar graphs indicating mean ± SD (*n* ≥ 3). Statistical analysis was performed using an unpaired, two-tailed Student's t-test, and *p*-values <0.05 were considered statistically significant.

### The paper explained

**PROBLEM:**

Mucolipidosis II (I-cell disease) is a lysosomal protein trafficking disorder in children accompanied by disabling skeletal alterations with unclear pathogenesis.

**RESULTS:**

Analyses of a mouse model of mucolipidosis II revealed that dysfunctional bone-forming osteoblasts and Il-6 driven increase in the number of functionally intact bone-resorbing osteoclasts rather than missorting of lysosomal hydrolases are responsible for the osteoporotic phenotype in the disease. Treatment with bisphosphonate led to an increased bone density and stabilization of the bones.

**IMPACT:**

Combinatorial histomorphometric and functional analyses of bone cells from a mouse model of mucolipidosis II provided novel insight into pathomechanisms and targets for therapeutic intervention which might also be applicable for other lysosomal disorders with skeletal defects.

## Author contributions

KKo, MA, TB and TS supervised the study, designed the experiments and wrote the manuscript. KKo, JMP, SCK, ES, MS, KKa, TO, AVF, RPM and TB conceived and performed experiments. PCL and MK collected and analysed data and RS is attending paediatrician and provided bone biopsy.

## For more information

1. OMIM:
2. http://omim.org/entry/252500
3. National MPS Society USA:
4. http://www.mpssociety.org/
5. Gesellschaft für Mucopolysaccharidosen e.V.:
6. http://www.mps-ev.de/

## Notes

As a service to our authors and readers, this journal provides supporting information supplied by the authors. Such materials are peer reviewed and may be re-organized for online delivery, but are not copy-edited or typeset. Technical support issues arising from supporting information (other than missing files) should be addressed to the authors.
