# Supplementary material for: Decreased bone formation and increased osteoclastogenesis cause bone loss in mucolipidosis II
Source: EMBO Mol Med. 2013 Oct 15;5(12):1871–86. doi: 10.1002/emmm.201302979 (PMC3914524; doi:10.1002/emmm.201302979)
Supplement: Supplementary file 1 [file emmm0005-1871-sd1.pdf]

Decreased bone formation and increased osteoclastogenesis cause bone loss in mucolipidosis II

Katrin Kollmann, Jan Malte Pestka, Sonja Christin K¸hn, Elisabeth Schöne, Michaela Schweizer, Kathrin Karkmann, Takanobu Otomo, Philip Catala-Lehnen, Antonio Virgilio Failla, Robert Percy Marshall, Matthias Krause, Rene Santer, Michael Amling, Thomas Braulke and Thorsten Schinke

Corresponding authors: Thomas Braulke and Thorsten Schinke, University Medical Center Hamburg-Eppendorf, Children's Hospital

Review timeline:

Submission date:	30 April 2013
Editorial Decision:	31 May 2013
Revision received:	24 August 2013
Editorial Decision:	06 September 2013
Revision received:	06 September 2013
Accepted:	09 September 2013

Transaction Report:

(Note: With the exception of the correction of typographical or spelling errors that could be a source of ambiguity, letters and reports are not edited. The original formatting of letters and referee reports may not be reflected in this compilation.)

Editor: Roberto Buccione

1st Editorial Decision 31 May 2013

Thank you for the submission of your manuscript to EMBO Molecular Medicine.

Unfortunately, in this case we experienced some difficulties in obtaining three timely evaluations. Since we cannot justify a further delay, I am sending the two consistent evaluations of Reviewers 2 and 3 at this time. I will forward Reviewer 1's delayed report, as soon as we are able to obtain it. If this report does arrive over the next few days and if it raises additional important issues that have to be addressed to support this study, these would also need to be taken into consideration in your revision. I would not ask you to consider further-reaching requests with respect to the current evaluations.

You will see that while both Reviewers are generally supportive of your work and underline its considerable potential interest, they also both raise a number of specific concerns that require your intervention and thus prevent us from considering publication at this time. I will not dwell into much detail, as the evaluations are detailed and self-explanatory.

Reviewer 2 feels that the gene expression section is poorly developed and suggests to either improve the informative value of the material supplied or to eliminate it altogether. I would encourage you to choose, and act upon the first solution. Reviewer 2 lists of number of other issues that require correction.

Reviewer 3 would like clarification with respect to the in vitro osteoclastogenesis studies using RANKL and vitamin D and wonders whether bone marrow stimulated with vitamin D in the absence of RANKL reveal defects in osteoclast formation. S/he would also like to know whether total or free RANKL was measured in the serum. Reviewer 3 notes that other cell types, in addition to osteoclasts, are a significant source of RANKL and would thus like you to identify the source of osteoclastogenesis-driving RANKL in your system. Reviewer 3 also lists of number of other important issues that require your action. I should mention that I agree that the title should be more reflective of the outcome (and compelling).

While publication of the paper cannot be considered at this stage, we would be pleased to consider a suitably revised submission, provided, however, that the Reviewers' concerns are fully addressed with additional experimental data where appropriate.

Please note that it is EMBO Molecular Medicine policy to allow a single round of revision only and that, therefore, acceptance or rejection of the manuscript will depend on the completeness of your responses included in the next, final version of the manuscript.

As you know, EMBO Molecular Medicine has a "scooping protection" policy, whereby similar findings that are published by others during review or revision are not a criterion for rejection. However, I do ask you to get in touch with us after three months if you have not completed your revision, to update us on the status. Please also contact us as soon as possible if similar work is published elsewhere.

I look forward to receiving your revised manuscript as soon as possible.

***** Reviewer's comments *****

Referee #2 (Comments on Novelty/Model System):

This paper contains a lot of data, which are presented in 9 figures that are generally very clear. The results of this study overturn the dogma that the skeletal abnormalities of MLII are due to secretion of a large excess of lysosomal enzymes and show that they are due to an increased number of osteoclasts. The results also indicate that the disease can be treated with alendronate. Whiule the medical impact is described as moderate simply because the disease is so rare, it will be of great importance to the patients and their families, as well as to patients with related lysosomal storage disorders.

Referee #2 (Remarks):

This is a high quality study, which seeks to explain why the mouse model of mucolipidosis II (I-cell disease) has an abnormal skeleton. The answer, somewhat surprisingly, is that it is due to the increase in the number of osteoclasts, and not to the secretion of excess lysosomal enzymes. The skeletal abnormalities were found treatable with alendronate. The approach may be extended to other lysosomal storage diseases manifesting themselves in skeletal abnormalities, and the treatment opportunities may be extended to human patients of this currently untreatable disease. But the paper, which is generally well written and beautifully illustrated, would benefit from some revisions. The suggestions are presented in the order in which they appear. Only the last one is major; the rest are quite minor.

p. 3. Mutation of the GNPTAB gene can result not only in MLII but also in MLIII, a milder disease, depending on the severity of the mutation (Bargal et al, Mol Genet Metab 88, 359-363, 2006; Cathey et al, Am J Med Genet 146A, 512-513, 2008, Cathey et al, J med Genet 47: 38-48, 2010).
p. 5. The expression "size-reduced" is not good English. Possible substitutions are "reduced in size" or "smaller".
p. 5. Please explain the term "dynamic histomorphometry".
p. 5. Could the term "crosslaps" please be defined in a couple of words? It may not be familiar to non-specialist readers.

p. 6 and fig. 3. What are the clear spaces seen in MLII as well as WT osteoclasts? At the magnification shown, they don't look any different from the clear spaces seen in the other types of MLII cells, where they are defined as lysosomal storage vacuoles.
p. 7. The calvariae and bone marrow were" differentiated ex vivo". By what method (please give a reference or a couple of words).
p. 8. The section about endocytosis and processing of radiolabeled arylsulfatase B is written in a confusing manner. I think that the point is that processing of endocytosed arylsulfatase B is delayed. But the reader is distracted by peripheral points such as "indicator protein" and an increase in surface Man 6-P receptors.

References: The chapter by Braulke et al in On Line MMBID (2013) appears not to be available yet. And in the text, this chapter is sometimes mistakenly referred to as "Braulke et al, 2012". It might be helpful to the reader to give the previous(2001) chapter in MMBID, by Kornfeld and Sly, or even to give some primary references to the earlier work by the groups of Kornfeld and von Figura.

Legends. Fig. 1 "growth retarded" would be better said as "retarded in growth".
Fig. 4D. Could the name of the bone be inserted - even though it may be stated in Methods.
Fig. 6. 6A. Could the word "intracellular" be inserted please-(Relative intracellular enzyme activity).

Pp. 9, 16, and supplementary material. The section on gene expression is poorly developed. The supplementary material tables present about 100 osteoblast genes the expression of which differs between MLII and wild type. However, only 2 were picked for confirmation by qRT-PCR, and one of those, Alpl, seems not to be on the list. Only IL6 was picked as an example of a deregulated cytokine though it is said that there are several on the list. To make the material more informative, it might be good to highlight (by using a different type, for example) those genes in Table 1 that are considered to be osteoblast marker proteins and those in Table 2 that are considered to encode inflammatory cytokines , and to justify the choice of genes for further study. It would also be helpful to give the full name of the genes. An alternative strategy would be to leave the supplementary tables for a subsequent paper, in which the subject would be developed more fully. In that case it would be necessay to change Fig 8 and 9 accordingly.

Referee #3 (Comments on Novelty/Model System):

This is an excellent and very comprehensive investigation into the mechanisms of bone loss in mucolipidosis II (MLII).

The authors have worked diligently to unravel a very complex mechanism of action and overall the study is very comprehensive, extensive and robust. I have one major criticism regarding some outmoded concepts of IL-6 action on bone and the prominence of osteoblasts as a source of RANKL, which call into question the strength of their conclusion that IL-6 is the central mediator of bone loss in this model. IL-6 is now considered to act though production of RANKL, not directly on osteoclasts. The source of RANKL in this model was not identified but several candidate cell types were not examined. Some relatively simple experiments quantifying RANKL production in WT and MLII mice from T and B cells, chondrocytes and osteocytes could potentially remedy this weakness.

Referee #3 (Remarks):

This study by Kollmann et al., is an extensive investigation of the mechanism of bone alterations in mucolipidosis II (MLII), a lysosomal storage disease characterized by lysosomal enzyme dysfunction. MLII leads to skeletal aberrations but the mechanisms involved are unclear.

Overall this is a robust and comprehensive study pairing biochemical, genetic and animal and translational human studies and culminating in a proof of concept study for the therapeutic efficacy of an anti-resorptive agent to alleviate bone loss in MLII Mice. While not surprising, given the

known actions of alendronate, the data are nonetheless important given the current clinical dilemma regarding if and how to treat the bone loss in these patients.
Interestingly, the data suggest a significant loss of bone mass as a consequence of increased osteoclastic bone resorption in vivo, although paradoxically osteoclast differentiation and resorptive activity ex vivo is demonstrated to be normal, despite defects in lysosomal enzymes. The data suggest an enhanced osteoclastogenic microenvironment as the underlying cause of elevated bone resorption and elevated IL-6 is postulated to be a central mediator of this bone loss.

Overall the studies are well designed and rigorous. My only major comments relate to the source of osteoclastogenic cytokines driving bone loss in this model and the proposed central role of IL-6 in this process, for which a direct cause-effect relationship is not clearly demonstrated, and does not fit well with current concepts of how IL-6 promotes bone resorption.

While it is understood that this is a rare genetic condition the reproducibility of data from a single MLII human patient is also somewhat of a concern.

Specific Points:
1)The in vitro osteoclastogenesis studies using whole bone marrow need some clarification as both vitamin D and RANKL are used as stimuli. High dose RANKL (as apparently used in this assay) tests the capacity of osteoclast precursors to differentiate and the data suggest no alteration in this aspect. However vitamin D is a stimulus for causing endogenous production of RANKL interrogating the responsiveness of the bone microenvironment to support osteoclast formation. Simultaneous use of the two stimuli abrogates the ability to probe the microenvironment. As the data show that the osteoclast differentiation step is unaffected by the disease, alterations in the expression of RANKL become a more important issue. Does bone marrow stimulated with vitamin D (without exogenous RANKL) reveal a defect in the ability to support osteoclast formation?

2) As acknowledged by the authors IL-6 is generally considered to promote osteoclastogenesis indirectly though induction of RANKL. However, no prominent cellular increases in RANKL were identified in the studies or in serum, a notoriously inaccurate site. Was total or free bioactive RANKL measured in serum? The authors have focused on osteoblasts as a source of RANKL and although the introduction proposes that osteoblasts are the dominant source of RANKL and OPG, these are somewhat dated concepts and recent studies have revealed that other cells such as osteocytes and chondrocytes are a significant source of RANKL (Nakashima et al. (2011) Nat Med 17(10): 1231-1234 and Xiong et al. (2011) Nat Med 17(10): 1235-1241) and under inflammatory conditions (a possibility suggested in this context by increased IL-6 production) T cells and B cells are a well-recognized source of RANKL. Several studies demonstrate that B cells are a major source of OPG (Li et al. (2007) Blood 109(9): 3839-3848). Furthermore, osteoblast production of RANKL is predominantly membrane associated and thus not assayed effectively by ELISA.
The authors should quantitate RANKL mRNA in osteoblasts and/or assess protein production from other cellular sources, to try and define the source of RANKL driving osteoclastogenesis in this system. One other potential explanation is involvement of a recently reported RANKL-independent cytokine from T cells that also promotes osteoblast IL-6 production (Rifas et al (2009) Arthritis Rheum 60(11): 3324-3335.).

Minor points:
The Title could be refined to better reflect the study outcomes. While I understand the point the authors are trying to make, technically there is no president for lysosomal enzyme missorting causing bone loss; hence the idea of an unexpected lack of involvement is somewhat confusing to the novice reader. Also the upswing in resorption is likely compounded by a decline in bone formation (Fig. 2D and 11). The importance of the later aspect is downplayed throughout the manuscript and its contribution should be more thoroughly discussed.

How were reference values obtained for DPD to compare the MLII patient to? There is no mention of control subjects.

The finding that alendronate did not reduce osteoclastogenesis (page 10) is not surprising as this drug works by inducing apoptosis in functional resorbing osteoclasts rather than targeting osteoclast differentiation. In fact, it's not clear that "osteoclastogenesis" per se was actually measured. The

failure to decrease osteoclast numbers/ BS (Figure 9) is puzzling but may potentially be explained by a simultaneous increase in bone surfaces resulting from diminished bone resorption.

1st Revision - authors' response 24 August 2013

We are happy that both reviewers shared our enthusiasm regarding the potential relevance of our findings. We have carried out additional experiments, which led to a new Figure 9 and additional panels included in Figures 5, 7 and 10 (the former Figure 9). Furthermore, the title was changed and Takanobu Otomo was included in the author list due to his contribution of B- and T-cell analyses (Figure 9).

We have modified the manuscript according to the reviewers' suggestions as follows:

Response to Reviewer #2:

1) p. 3. Mutation of the GNPTAB gene can result not only in MLII but also in MLIII, a milder disease, depending on the severity of the mutation (Bargal et al, Mol Genet Metab 88, 359-363, 2006; Cathey et al, Am J Med Genet 146A, 512-513, 2008, Cathey et al, J med Genet 47: 38-48, 2010).

The reviewer is right. As suggested, we added the criteria of different mutations (nonsense, frameshift, splice site alterations vs missense mutations) to described the differentially affected patients (MLII alpha/beta and MLIII alpha/beta, respectively). p3

2) p. 5. The expression "size-reduced" is not good English. Possible substitutions are "reduced in size" or "smaller".

We have changed our manuscript according to the reviewer´s suggestion.

3) p. 5. Please explain the term "dynamic histomorphometry".

Dynamic histomorphometry is a standard technique to quantify bone formation parameters based on dual injection of flourochromes binding to mineralized bone. Since we agree with the reviewer that this method is probably unknown to many readers outside the bone field, we have briefly explained it in the Results section of the revised manuscript. p5/6

4) p. 5. Could the term "crosslaps" please be defined in a couple of words? It may not be familiar to non-specialist readers.

This is a similar issue, and we have explained this in greater detail in the revised version of the manuscript. p6, 1st paragraph

© EMBO

5) p. 6 and fig. 3. What are the clear spaces seen in MLII as well as WT osteoclasts? At the magnification shown, they don't look any different from the clear spaces seen in the other types of MLII cells, where they are defined as lysosomal storage vacuoles.

The osteoclast is a highly specialized cell type with many unique properties. It is a giant multinucleated cell forming a ruffled border, and it contains a large number of intracellular organelles (Boyle et al., Nature, 2003). In addition, the cytoplasm of osteoclasts is characterized by the presence of large numbers of vacuoles and lucent bodies near the ruffled border. These cytoplasmic vacuoles were first described by Jackson in 1904 through the use of light microscopy. About 60 years later,this was confirmed by electron microscopy and the use of aldehydes as fixatives in preparation of bone tissue (Scott, 1967; Schenk et al., 1967). Subsequent observations by electron microscopy have shown that the cytoplasmic bodies and vacuoles in osteoclasts contain acid phosphatase (Dory et al., 1968; Lucht, 1971). Most importantly however, and this has already been stated in the original manuscript, osteoclasts from wild-type and MLII mice both displayed these typical characteristics and were morphologically indistinguishable from each other, in contrast to osteoblasts or osteocytes.

We have summarized these data in the revised manuscript. p6

6) p. 7. The calvariae and bone marrow were" differentiated ex vivo". By what method (please give a reference or a couple of words).

We have now stated more clearly how the cells were isolated and cultured in the revised version of the manuscript. p7, p9, p 17-19

7) p. 8. The section about endocytosis and processing of radiolabeled arylsulfatase B is written in a confusing manner. I think that the point is that processing of endocytosedarylsulfatase B is delayed. But the reader is distracted by peripheral points such as "indicator protein" and an increase in surface Man 6-P receptors.

We have explained this issue more carefully in the revised version of the manuscript. p9

8) References: The chapter by Braulke et al in On Line MMBID (2013) appears not to be available yet. And in the text, this chapter is sometimes mistakenly referred to as "Braulke et al, 2012". It might be helpful to the reader to give the previous(2001) chapter in MMBID, by Kornfeld and Sly, or even to give some primary references to the earlier work by the groups of Kornfeld and von Figura.

We prefer the actual version of this reference from 2013 because it contains the latest findings and new information for MLII. The new chapter by Braulke, Raas-Rothschild, Kornfeld is an update of the previous chapter by Kornfeld and Sly.

The chapter is available online using the following link:

http://www.ommbid.com/OMMBID/the_online_metabolic_and_molecular_bases_of_inherited_disease/b/abstract/part16/ch138

9) Legends. Fig. 1 "growth retarded" would be better said as "retarded in growth".

We agree and have changed the manuscript accordingly.

10) Fig. 4D. Could the name of the bone be inserted - even though it may be stated in Methods.

This was an iliac crest biopsy, which is now stated in the Figure legend as well. p7, p30

11) Fig. 6. 6A. Could the word "intracellular" be inserted please-(Relative intracellular enzyme activity).

We have changed the legend according to the reviewer´s suggestion. p31

12) Pp. 9, 16, and supplementary material. The section on gene expression is poorly developed. The supplementary material tables present about 100 osteoblast genes the expression of which differs between MLII and wild type. However, only 2 were picked for confirmation by qRT-PCR, and one of those, Alpl, seems not to be on the list. Only IL6 was picked as an example of a deregulated cytokine though it is said that there are several on the list. To make the material more informative, it might be good to highlight (by using a different type, for example) those genes in Table 1 that are considered to be osteoblast marker proteins and those in Table 2 that are considered to encode inflammatory cytokines , and to justify the choice of genes for further study. It would also be helpful to give the full name of the genes. An alternative strategy would be to leave the supplementary tables for a subsequent paper, in which the subject would be developed more fully. In that case it would benecessay to change Fig 8 and 9 accordingly.

We fully agree with the reviewer that the previous way of presenting the expression data was not very informative. Since the full data sets have been submitted to the GEO database, we have now removed the supplementary tables 1 and 2 and instead inserted a new panel into Figure 7, where we only show the well-established osteoblast differentiation markers(7C) and the three differentially expressed genes with a known effect on osteoclastogenesis (7D). The qRT-PCR expression analysis shown in Fig. 7B now also contains *Bglap* (the gene encoding osteocalcin), since we thereby analyzed expression of the three most common osteoblast markers (*Alpl, Ibsp, Bglap*), and as shown in the new Fig. 7C, these were all identified as differentially expressed in the genome-wide expression analysis. Regarding IL6 we have to admit that it is still only a candidate gene potentially contributing to increased osteoclastogenesis in MLII mice, and we would like to refer to our response to the second comment of reviewer #3 for additional information.

According to the changes and additional experiments, we have rewritten the Results section p9/10

Response to Reviewer #3:

1)The in vitro osteoclastogenesis studies using whole bone marrow need some clarification as both vitamin D and RANKL are used as stimuli. High dose RANKL (as apparently used in this assay) tests the capacity of osteoclast precursors to differentiate and the data suggest no alteration in this aspect. However vitamin D is a stimulus for causing endogenous production of RANKL interrogating the responsiveness of the bone microenvironment to support osteoclast formation. Simultaneous use of the two stimuli abrogates the ability to probe the microenvironment. As the data

show that the osteoclast differentiation step is unaffected by the disease, alterations in the expression of RANKL become a more important issue. Does bone marrow stimulated with vitamin D (without exogenous RANKL) reveal a defect in the ability to support osteoclast formation?

We fully agree with the reviewer and have performed the osteoclastogenic differentiation assays with bone marrow cells that were only treated with vitamin D. These data have been included in Figure 5D, and they essentially confirm our previous conclusion. p8

2) As acknowledged by the authors IL-6 is generally considered to promote osteoclastogenesis indirectly though induction of RANKL. However, no prominent cellular increases in RANKL were identified in the studies or in serum, a notoriously inaccurate site. Was total or free bioactive RANKL measured in serum? The authors have focused on osteoblasts as a source of RANKL and although the introduction proposes that osteoblasts are the dominant source of RANKL and OPG, these are somewhat dated concepts and recent studies have revealed that other cells such as osteocytes and chondrocytes are a significant source of RANKL (Nakashima et al. (2011) Nat Med 17(10): 1231-1234 and Xiong et al. (2011) Nat Med 17(10): 1235-1241) and under inflammatory conditions (a possibility suggested in this context by increased IL-6 production) T cells and B cells are a well-recognized source of RANKL. Several studies demonstrate that B cells are a major source of OPG (Li et al. (2007) Blood 109(9): 3839-3848). Furthermore, osteoblast production of RANKL is predominantly membrane associated and thus not assayed effectively by ELISA.

The authors should quantitate RANKL mRNA in osteoblasts and/or assess protein production from other cellular sources, to try and define the source of RANKL driving osteoclastogenesis in this system. One other potential explanation is involvement of a recently reported RANKL-independent cytokine from T cells that also promotes osteoblast IL-6 production (Rifas et al (2009) Arthritis Rheum 60(11): 3324-3335.).

Given the importance of this particular comment, we have performed several additional experiments that were included into the revised version of the manuscript. More specifically, we have determined *Rankl* expression in T and B cells from wild-type and MLII mice, where we did not detect significant differences (Fig. 9A). We have also quantified the number of bone marrow T cells, but there was no difference between wild-type and MLII mice either (Fig. 9B). In addition, we analyzed expression of *Tnsf11*, *Tnfrsf11b* and *Il6* in terminally differentiated primary calvarial osteoblasts (d30 of differentiation) and in primary chondrocytes (d20 of differentiation) from wild-type and MLII mice. While there was no difference in the expression of the Rankl/Opg-encoding genes, *Il6* was again markedly over-expressed in the MLII cell types (Fig. 9C/D).We fully agree with both reviewers that it is debatable, whether increased IL-6 production in MLII mice is indeed responsible for their excessive osteoclastogenesis. The probably best way to address this possibility*in vivo*would be the generation of *Il6*-deficient MLII mice, which is however beyond the scope of our manuscript.We would however like to point out that there areotherstudies demonstrating a Rankl-independent pro-osteoclastogenic effect of IL6, both *in vitro* and *in vivo* (Kudo et al, 2003; de Benedetti et a., 2006). p11/12

3) The Title could be refined to better reflect the study outcomes. While I understand the point the authors are trying to make, technically there is no president for lysosomal enzyme missorting causing bone loss; hence the idea of an unexpected lack of involvement is somewhat confusing to the novice reader. Also the upswing in resorption is likely compounded by a decline in bone formation (Fig. 2D and 11). The importance of the later aspect is downplayed throughout the manuscript and its contribution should be more thoroughly discussed.

We have modified the title accordingly and discussed the relevance of decreased bone formation in the context of our findings.

4) How were reference values obtained for DPD to compare the MLII patient to? There is no mention of control subjects.

Since it is generally a problem to obtain age-matched control subjects for pediatric diseases we compared the values to the children reference ranges reported in the literature. We would however like to emphasize that these reference ranges are in full agreement with the values obtained in our Department of Clinical Chemistry, where DPD levels measured in other pediatric diseases are typically below 20 nmol/nmolcreatinine.

5) The finding that alendronate did not reduce osteoclastogenesis (page 10) is not surprising as this drug works by inducing apoptosis in functional resorbing osteoclasts rather than targeting osteoclast differentiation. In fact, it's not clear that "osteoclastogenesis" per se was actually measured. The failure to decrease osteoclast numbers/ BS (Figure 9) is puzzling but may potentially be explained by a simultaneous increase in bone surfaces resulting from diminished bone resorption.

We agree with the reviewer that the failure to decrease osteoclast surface in MLII mice is kind of puzzling and we do not have a sufficient explanation for this observation yet. What is remarkable however is that similar findings wereobtained for the osteoblast surface and bone formation rate, both being reduced by alendronate in wildtype mice, but not in MLII mice. These data have been integrated into the revised version of the manuscript (Fig. 10C), and they suggest that the pro-osteoclastogenic signals coming from MLII osteoblasts are still produced upon anti-resorptive treatment. Regardless of these potentially interesting findings however, we still believe that the most important result is that alendronate can significantly increase bone mass in MLII mice. Given the fact that MLII is a disease, where most cell types are negatively affected, it is quite important that one aspect of the disease can be treated by blocking one specific cell type that is still intact, but numerically increased in MLII. p12

2nd Editorial Decision 06 September 2013

Thank you for the submission of your revised manuscript to EMBO Molecular Medicine. We have now received the enclosed reports from the two Reviewers that were asked to re-assess it. As you will see below, the Reviewers are now globally supportive with some final requests, and I am pleased to inform you that we will be able to accept your manuscript pending the following final amendments (which will be dealt with editorially):

1) Reviewer 2 feels that an alternative explanation is possible for the outcome of the experiment depicted in Fig.6. I would encourage you to consider this in your manuscript.

2) Both Reviewers suggest e few minor corrections in the text. Although this could have been taken care of at the proof stage, you might as well apply these corrections now.

3) As per our Author Guidelines, the description of all reported data that includes statistical testing must state the name of the statistical test used to generate error bars and P values, the number (n) of independent experiments underlying each data point (not replicate measures of one sample), and the actual P value for each test (not merely 'significant' or 'P < 0.05').

Please provide the new manuscript without coloured lettering (no longer needed).

I look forward to reading a new revised version of your manuscript as soon as possible and in any

case, within two weeks.

***** Reviewer's comments *****

Referee #2 (Comments on Novelty/Model System):

This is a very impressive paper. It is of excellent quality, and offers a new way to look at the skeletal abnormalities in Mucolipidosis II. A treatment is suggested, which might be applicable to human patients and which also might be useful for other genetic diseases with similar skeletal defects.

Referee #2 (Remarks):

I would disagree with the authors' interpretation of one result. On p. 9, it is stated that the internalization of more [125I]-labeled ASB precursor by MLII fibroblasts suggests that "lysosomal delivery along the endocytic pathway is affected in MLII cells". I believe that there is another explanation for the higher uptake of [125I]-ASB by MLII cell, and it does not involve any putative defect in the MLII lysosomal pathway. In normal cells, the radiolabeled ASB has to compete with endogenous Man6P proteins (that had been secreted) for binding to the Man6P receptor on the cell surface and to other binding proteins (if such exist) along the pathway. In MLII cells, the radiolabeled ASB has no such competition.

Suggestion for two trivial corrections:

p. 3 "the majority of patients ... has" should be "the majority of patients ... HAVE".
p. 23 "Impact"
....targets for therapeutic intervention which might ALSO be applicable to other lysosomal disorders with skeletal defects".

Referee #3 (Comments on Novelty/Model System):

This revision has adequately addressed my previous concerns.

Referee #3 (Remarks):

This revision has adequately addressed my previous concerns.One typo in the Figure 10C legend "per bne surface" to be fixed in the proof.

2nd Revision - authors' response 06 September 2013

We thank you and the reviewers that are almost pleased with the requested revisions of the manuscript. The minor requests for further amendments were considered as follows:

Reviewer #2:

I would disagree with the authors' interpretation of one result. On p. 9, it is stated that the internalization of more [125I]-labeled ASB precursor by MLII fibroblasts suggests that "lysosomal delivery along the endocytic pathway is affected in MLII cells". I believe that there is another explanation for the higher uptake of [125I]-ASB by MLII cell, and it does not involve any putative defect in the MLII lysosomal pathway. In normal cells, the radiolabeled ASB has to compete with endogenous Man6P proteins (that had been secreted) for binding to the Man6P receptor on the cell surface and to other binding proteins (if such exist) along the pathway. In MLII cells, the radiolabeled ASB has no such competition.

This is an interesting argument, which is, however, rather unlikely for the following reasons:

Firstly, the cells were washed and preincubated in serum-free medium prior addition of the [125I]-labeled ASB precursor excluding an accumulation of endogenous Man6P-containing ligands, which might significantly affect the amounts of [125I]-labeled ASB precursor during the 20 min internalization period. Secondly, the amounts of 300 kDa Man6P receptor (MPR300) at the cell surface of both human MLII (I-cell) fibroblasts (Braulke et al., 1992) and mice MLII fibroblasts (Sandra Markmann and Katrin Kollmann, unpublished results) are increased 2- to 3.0-fold in comparison with healthy control and wild-type cells, respectively. Since the increased concentration of MPR300 at the cell surface results in a proportional increase of internalized Man6P-containing ligands (Braulke et al., 1989), the 3.5-fold increase in internalized [125I]-labeled ASB precursor in MLII osteoblasts is most likely due to similarly increased number of MPR300 at the cell surface. Thirdly, about 25% of all MPR300 localized at the cell surface are occupied at steady state with endogenous Man6P-containing ligands (Braulke et al., 1987) which also excluded competitive mechanisms. Furthermore, the MPR46 also localized at the cell surface, is not able to internalize Man6P-containing ligands (Stein et al., 1987).

References:

Braulke T, Tippmer S, Matzner U, Gartung C, von Figura K (1992) Mannose 6-phosphate/insulin-like growth factor II receptor in I-cell disease fibroblasts: increased synthesis and defective regulation of cell surface expression. Biochim Biophys Acta 1138: 334-342

Braulke T, Tippmer S, Neher E, von Figura K (1989) Regulation of the mannose 6-phosphate/IGF II receptor expression at the cell surface by mannose 6-phosphate, insulin like growth factors and epidermal growth factor. EMBO J 8: 681-686

Braulke T, Gartung C, Hasilik A, von Figura K (1987) Is movement of mannose 6-phosphate-specific receptor triggered by binding of lysosomal enzymes? J Cell Biol 104: 1735-1742

Stein M, Zijderhand-Bleekemolen JE, Geuze H, Hasilik A, von Figura K (1987) Mr 46,000 mannose 6- phosphate specific receptor: its role in targeting of lysosomal enzymes. EMBO J 6: 2677-2681

Therefore, we like to leave the sentence (p8): Incubation of wild-type and MLII osteoblasts with [125I]-labeled M6P-containing arylsulfatase B (ASB) showed that MLII osteoblasts internalized 3.5-fold higher amounts of [125I]-labeled ASB precursor in an M6P-dependent manner compared to wild-type cells suggesting that the lysosomal delivery along the endocytic pathway is affected in MLII cells. as it is.

We added the following suggested corrections to the text

p. 3 "the majority of patients... HAVE…".

p. 23 "Impact" ....targets for therapeutic intervention which might ALSO be applicable to other lysosomal disorders with skeletal defects".

Fig. 10C legend "per bone surface"

Additionally, we added the actual p-value and the number of independent experiments (n) for each significant test to the figure legend. We always performed the independent student’s Ttest as already stated in the method section and now additionally in the figure legends.

We thank again for the helpful comments and suggestions and hope that the final amendments make the ma
